# Uncovering the out-of-plane nanomorphology of organic photovoltaic bulk heterojunction by GTSAXS

**DOI:** 10.1038/s41467-021-26510-6

**Published:** 2021-10-28

**Authors:** Xinxin Xia, Tsz-Ki Lau, Xuyun Guo, Yuhao Li, Minchao Qin, Kuan Liu, Zeng Chen, Xiaozhi Zhan, Yiqun Xiao, Pok Fung Chan, Heng Liu, Luhang Xu, Guilong Cai, Na Li, Haiming Zhu, Gang Li, Ye Zhu, Tao Zhu, Xiaowei Zhan, Xun-Li Wang, Xinhui Lu

**Affiliations:** 1 Department of Physics, The Chinese University of Hong Kong, New Territories, Hong Kong, 999077 China; 2 Department of Applied Physics, Research Institute for Smart Energy, The Hong Kong Polytechnic University, Hung Hom, Hong Kong, China; 3 Department of Electronic and Information Engineering, Research Institute for Smart Energy (RISE), The Hong Kong Polytechnic University, Hung Hom, Kowloon, Hong Kong, China; 4 Center for Chemistry of High-Performance & Novel Materials, Department of Chemistry, Zhejiang University, Hangzhou, 310027 Zhejiang China; 5 Spallation Neutron Source Science Center, Dongguan, 523803 China; 6 Institute of High Energy Physics, Chinese Academy of Sciences, Beijing, 100049 China; 7 National Facility for Protein Science in Shanghai, Zhangjiang Laboratory, Shanghai Advanced Research Institute, Chinese Academy of Science, No.333, Haike Road, Shanghai, 201204 People’s Republic of China; 8 Beijing National Laboratory for Condensed Matter Physics and Institute of Physics, Chinese Academy of Sciences, Beijing, 100190 China; 9 School of Materials Science and Engineering, Peking University, Beijing, 100871 China; 10 Department of Physics and Center for Neutron Scattering, City University of Hong Kong, Kowloon, Hong Kong, China

**Keywords:** Devices for energy harvesting, Solar cells, Characterization and analytical techniques

## Abstract

The bulk morphology of the active layer of organic solar cells (OSCs) is known to be crucial to the device performance. The thin film device structure breaks the symmetry into the in-plane direction and out-of-plane direction with respect to the substrate, leading to an intrinsic anisotropy in the bulk morphology. However, the characterization of out-of-plane nanomorphology within the active layer remains a grand challenge. Here, we utilized an X-ray scattering technique, Grazing-incident Transmission Small-angle X-ray Scattering (GTSAXS), to uncover this new morphology dimension. This technique was implemented on the model systems based on fullerene derivative (P3HT:PC_71_BM) and non-fullerene systems (PBDBT:ITIC, PM6:Y6), which demonstrated the successful extraction of the quantitative out-of-plane acceptor domain size of OSC systems. The detected in-plane and out-of-plane domain sizes show strong correlations with the device performance, particularly in terms of exciton dissociation and charge transfer. With the help of GTSAXS, one could obtain a more fundamental perception about the three-dimensional nanomorphology and new angles for morphology control strategies towards highly efficient photovoltaic devices.

## Abstract (web summary)

Morphology of organic thin film, including the in-plane and out-of-plane directions, plays a crucial role in determining the performance of organic solar cells, yet the characterisation is challenging for the out-of-plane direction. Here, the authors use GTSAXS to uncover the nanomorphology in this dimension, and show how it affects exciton dissociation and charge transfer.

## Introduction

Organic photovoltaics (OPVs) have gained much attention owing to their potential to offer low-cost, high-performance, and flexible devices^1^. To cope with the intrinsic strong exciton-binding energy and short carrier diffusion length, OPVs usually employ the bulk heterojunction (BHJ) device structure. Organic p-type (donor) and n-type (acceptor) semiconducting materials are mixed in the active layer to create rich donor/acceptor (D/A) interfaces for exciton dissociation. Concomitantly, complex bulk morphology, in terms of molecular packing and phase separation, is generated, critically influencing exciton dissociation, charge transfer, and transport behaviors, and thus the overall device performance. The importance of bulk morphology for the OPV device performance has been well recognized and intensively researched^2–7^ along with the progressing of the recording device power conversion efficiency (PCE) over 18%^8^.

In general, the bulk morphology of an OPV active layer contains both the molecular level and nanoscale structural information. Grazing incidence wide-angle X-ray scattering (GIWAXS) is often implemented to investigate molecular-level structural information in terms of crystallinity, lattice constants, and crystalline domain orientations^9^. To acquire the nanoscale structural information, grazing incidence small-angle X-ray scattering (GISAXS)^10^ and resonant soft X-ray scattering (RSoXS)^11,12^ are often employed. In 2013, Liao et al.^13^ carried out GISAXS measurements to study the effect of additive 1,8-diiodooctane (DIO) on the phase separation of blend PCPDTBT:PC_71_BM films and a fractal-like network model was used to fit the GISAXS horizontal profiles^13^. Our group utilized the combination of GIWAXS and GISAXS to understand the morphology compatibility rule of a series of ternary BHJ systems^5,6^. In 2012, the miscibility and domain purity of PTB7:PC_71_BM thin films with DIO was first revealed using RSoXS by Ade’s group^11^. The same group recently correlated the total scattering intensity with the Flory–Huggins interaction parameter (*χ*) of various OPV systems, which helped predict the attainable fill-factor^7^. However, the quantitative nanostructure exploited by GISAXS and RSoXS is so far along the in-plane (IP) direction with respect to the thin film surface. Although GISAXS pattern contains the out-of-plane (OOP) nanomorphology information, the scattering features are disturbed by the so-called Yoneda peak owing to reflection and refraction effects^14^. Note here, the IP and OOP directions are referring to the lateral and normal directions with respect to the thin film surface, respectively. More complicated distorted-wave Born approximation (DWBA) needs to be accounted for data fitting^10^, especially challenging when the feature of interest is in the vicinity of Yoneda peak. On the other hand, there is a powerful technique–X-ray reflectivity (XRR), that can probe thin-film structural information along the surface normal directions^15^, such as film thickness, interfacial roughness, and material distribution, however, it is still not straightforward to extract the OOP nanomorphology within the films by XRR.

Nevertheless, the importance of the OOP nanomorphology has been noticed for years. In 2002, Huck et al.^16^ improved the quantum efficiency of the device by controlling the vertical phase separation in the organic BHJ active layer of PFB:P8BT. Moreover, Campoy-Quiles et al.^17^ identified a generic phase separation order, whereby the polymer crystallized first and was followed by the diffusion of PC_61_BM, forming lateral and vertical domains. In 2009, Yang et al.^18^ confirmed the existence of vertical phase separation using a P3HT:PC_71_BM blend film, in which P3HT appeared at the air-organic interface and PC_71_BM appeared at the substrate-organic interface. They proposed to use an inverted device structure to solve this inhomogeneity issue. Since then, various studies have employed inverted structures for efficient charge collection. Given the critical effect of OOP morphology, several studies have employed various methods to control it, for instance, by adding nanowires to the active layer^19^ or by using different interfaces^20^. Thus, the control of OOP nanomorphology plays an important role in the optimization of the device performance, however, still at an empirical level, lack of viable quantitative characterization methods.

In this work, to reveal the OOP nanomorphology of organic BHJ active layers, an X-ray scattering technique called grazing-incident transmission small-angle X-ray scattering (GTSAXS) was employed^21^. This technique allows the detection of scattering under the surface horizon of the film which, unlike GISAXS, could be modeled readily using modified simple Born approximation^10^. We first demonstrate the application of the GTSAXS method on a prototypical OPV system, P3HT:PC_71_BM^18,22^, by comparing the results side-by-side with those of GISAXS. It is evident that GTSAXS could extract the IP structural information as GISAXS does, whereas concurrently providing the OOP structural information. As a result, the statistical three-dimensional (3D) nanomorphology of the OPV BHJ thin film was detected quantitatively by GTSAXS for the first time. Then, we generalize the applicability of this method to two typical non-fullerene (NF) acceptor-based systems, PBDB-T:ITIC^23,24^ and PM6:Y6^25^ fabricated with different processing conditions. The correlation between the 3D nanomorphology and device performance is discussed and established. It is suggested that the nanomorphology in the OOP direction should be one of the vital factors determining the device performance. This work opens a new dimension of OPV BHJ morphology—the OOP nanomorphology, providing insights into the future accurate morphology control towards highly efficient devices. Furthermore, the proposed analysis scheme can be also readily migrated to study the inner 3D nanostructure of other functional thin film systems beyond OPV, such as polymer materials, nanofiltration membranes, quantum dot, and perovskite thin films.

## Results

### Direct comparison of GISAXS and GTSAXS

**Fig. 1:**
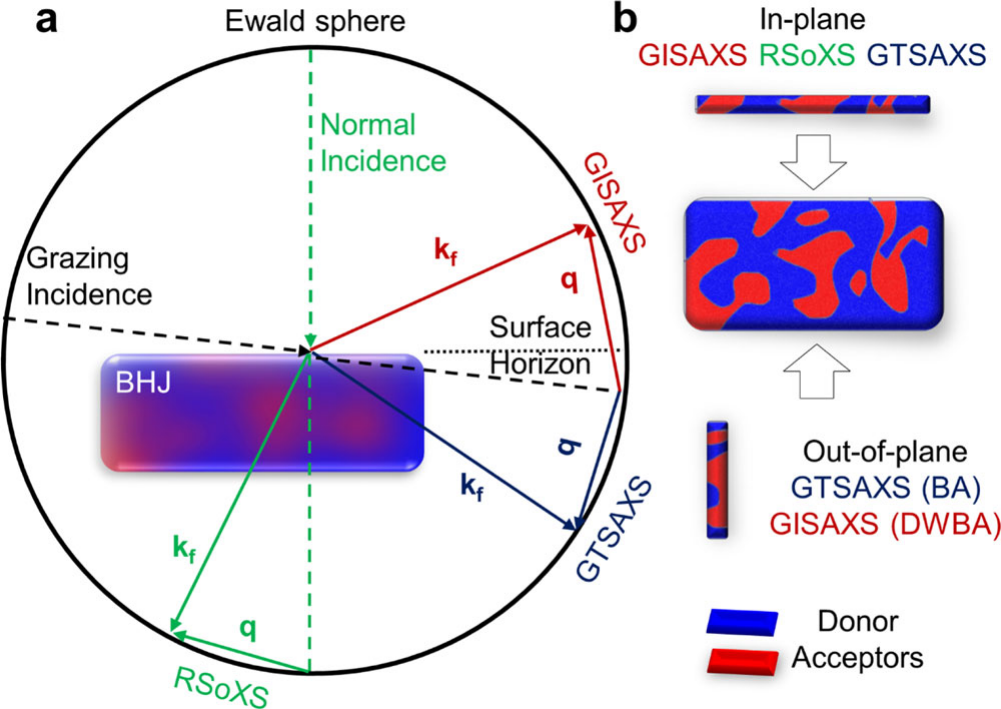
Comparison of GTSAXS, GISAXS, and RSoXS. **a** The experimental geometry comparison of GTSAXS, GISAXS, and RSoXS and **b** the illustration of the capability and the approximation of each technique to quantitatively extract the in-plane and out-of-plane nanomorphology.

The experimental geometries of GTSAXS, GISAXS, and RSoXS are illustrated in Fig. 1a to highlight their key differences in thin-film structure probing. First, GTSAXS and GISAXS both employ a grazing-incident beam^10,21^, whereas RSoXS is typically performed in the normal incidence^12^. Second, GISAXS collects the reflected outgoing beam, whereas RSoXS and GTSAXS both collect the transmitted outgoing beam. Specifically, RSoXS collects the signal transmitted through the backside of the thin film sample, but GTSAXS collects the signal exited from the front edge by aligning the cleaved front edge of the thin film sample at the center of the goniometer stage (Supplementary Fig. 1, details in method section). To eliminate potential edge effects, one needs to carefully cleave the sample at the center region, in the same way as in cross-sectional scanning electron microscopy (SEM), to form a clean edge, as illustrated in Supplementary Fig. 2a, b. In principle, the orientation of the structure ordering probed is determined by the direction of the scattering wave vector ${{{{{\bf{q}}}}}}={{{{{{\bf{k}}}}}}}_{f}-{{{{{{\bf{k}}}}}}}_{i}$. The **q** vector of GTSAXS and GISAXS have both parallel and vertical components with respect to the surface, thus capable of providing both the IP and OOP nanomorphology. In contrast, RSoXS only offers IP nanomorphology since the **q** vector mainly falls in the IP direction. Therefore, in the following, we focus on comparing the scattering results of GTSAXS and GISAXS side-by-side.

**Fig. 2:**
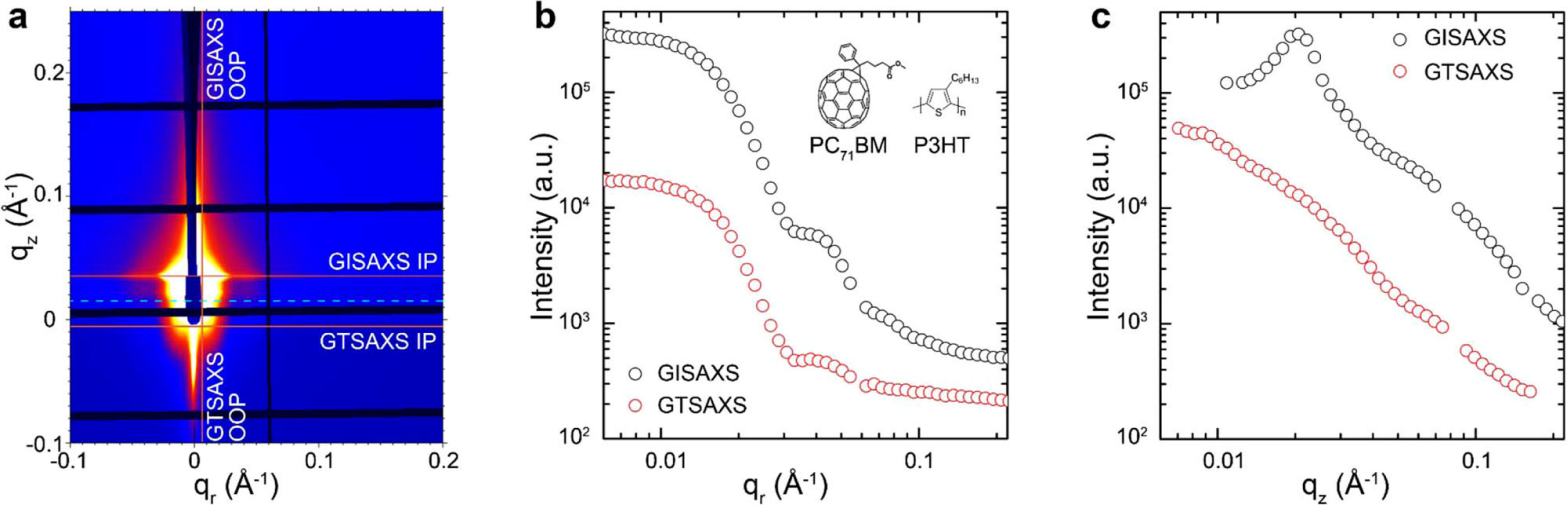
GISAXS and GTSAXS of fullerene-based BHJ. **a** The 2D-scattering pattern of P3HT:PC_71_BM thin film measured at an incidence angle of 0.15^o^. The orange lines highlight the positions to perform the intensity linecuts. The cyan dotted line illustrates the position of the surface horizon. The **b** IP and **c** OOP intensity profiles were extracted in the GISAXS region (black) and in the GTSAXS region (red). The inset of **b** shows the chemical structure of P3HT and PC_71_BM.

To demonstrate the capability of GTSAXS in extracting both the IP and OOP nanomorphology of the OPV active layer, we choose a model system of P3HT:PC_71_BM, whose IP nanomorphology was extensively studied by GISAXS and RSoXS previously^14,26–31^. In principle, by impinging the grazing-incident light at the front edge of the thin film sample, both GISAXS and GTSAXS signals could appear in the same two-dimensional (2D) scattering pattern, which is separated by the surface horizon^21^. Figure 2a shows a 2D-scattering pattern of a P3HT:PC_71_BM film measured with front edge X-ray impinging at an incidence angle of 0.15^o^. The horizontal cyan dashed line at **q**_*z*_ ≈ 0.016 Å^−1^ indicates the position of the surface horizon. The GISAXS signal appears above the surface horizon while the GTSAXS signal appears below.

First, we performed the horizontal linecuts above and below the surface horizon to extract the GISAXS and GTSAXS intensity profiles respectively, both of which should offer the IP nanomorphology information of the P3HT:PC_71_BM film, as shown in Fig. 2b. To enhance the overall intensity of the scattering profiles, the GISAXS IP profile is often obtained at the Yoneda peak position^10^, which is observed at the scattering angle ${\alpha }_{f}={\alpha }_{i}+{\alpha }_{{{{{{\rm{ct}}}}}}}$, where ${{{{{{\rm{\alpha }}}}}}}_{i}$ is the incident angle and ${{{{{{\rm{\alpha }}}}}}}_{{{{{{\rm{ct}}}}}}}$ is the critical angle of the sample. The critical angle of the P3HT:PC_71_BM film is calculated to be 0.11° at 12 keV. In contrast, the GTSAXS IP profile is extracted at **q**_*z*_ = −0.008 Å^−1^ near the minimum achievable **q**_*z*_, determined by the size of the beamstop. As manifested in Fig. 2b, GISAXS and GTSAXS IP profiles highly resemble each other, except for a relatively higher intensity of the GISAXS signal owing to the surface enhancement effect. Such a resemblance demonstrates that GTSAXS is comparably capable of revealing the IP nanomorphology as GISAXS does.

In contrast, the OOP intensity profiles in the GISAXS and GTSAXS regions are noticeably different. As illustrated in Fig. 2a, the vertical linecuts are performed at the minimum achievable **q**_*r*_ position (**q**_*r*_ = 0.006 Å^−1^), which is also limited by the beamstop. The surface horizon separates the boundary of GISAXS and GTSAXS OOP profiles. The GISAXS OOP profile exhibits a pronounced Yoneda peak at **q**_*z*_ ~ 0.03 Å^−1^. Owing to the strong intensity of Yoneda peak, the scattering features from the sample in the **q**_*z*_ range between ~ 0.01 and ~ 0.06 Å^−1^ are totally shadowed. Even the powerful DWBA can barely draw meaningful structural information in that region. Remarkably, the GTSAXS OOP profile, apparently free of Yoneda peak, presents characteristic intensity variation versus **q**, a common scattering feature of nanoscale PCBM clusters^31^. The IP and OOP GTSAXS/GISAXS profiles for bare Si substrate are presented in Supplementary Fig 3, which show typical **q**-dependent intensity decay profiles of diffuse scattering, which is obviously different from those profiles of thin-film samples, further confirming that GTSAXS is mainly detecting the structural features from the thin films.

### Extraction of quantitative 3D nanomorphology information

**Fig. 3:**
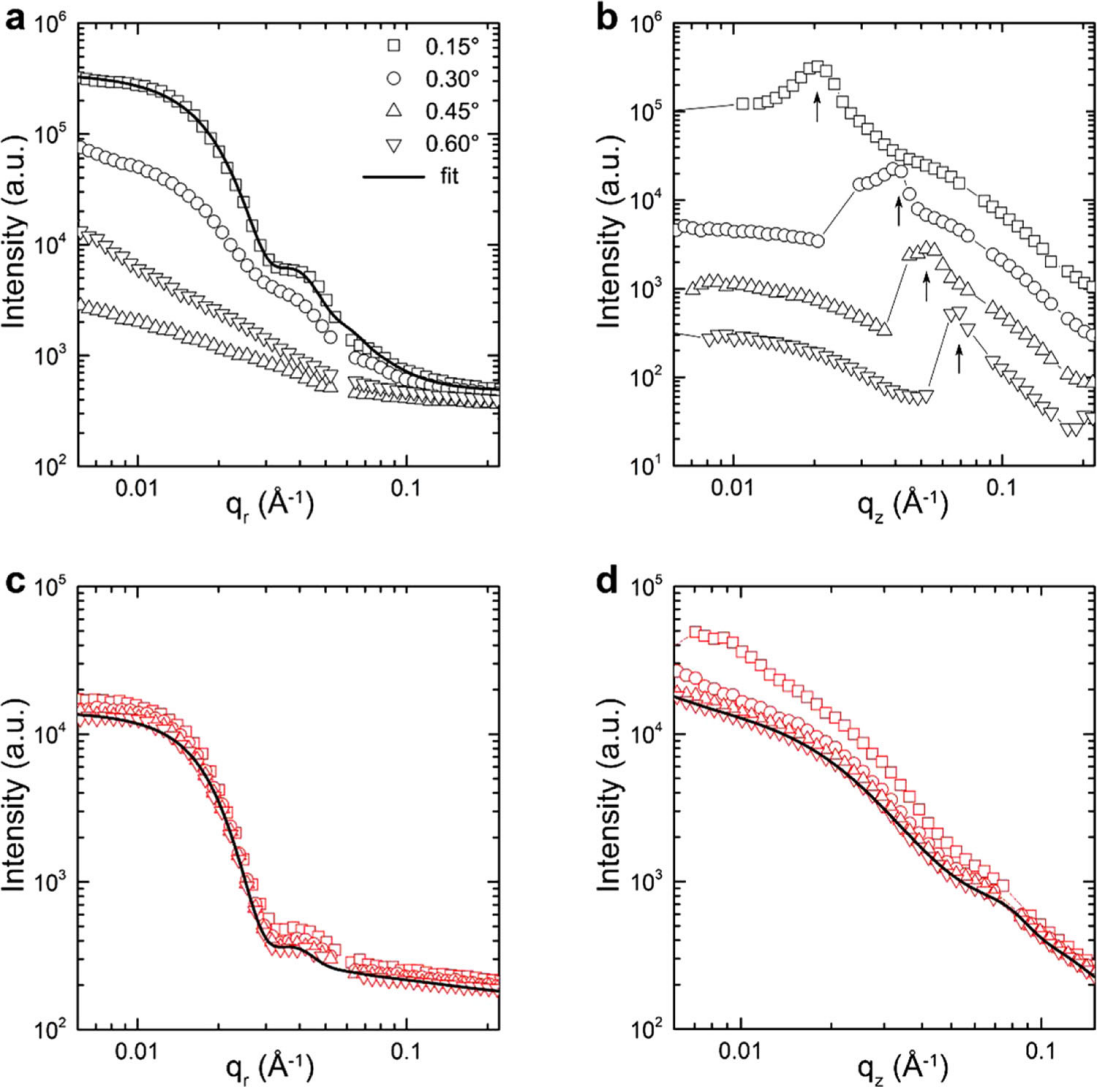
Formalism of GTSAXS measurements. The **a** IP and **b** OOP intensity profiles were extracted in the GISAXS at different incident angles. The **c** IP and **d** OOP intensity profiles were extracted in the GTSAXS region at different incident angles. Only the profiles in **b** are shifted for better visualization. The arrows in **b** highlight the Yoneda peak positions. The solid lines are model fitting to the data.

To set up a standard experimental protocol to extract quantitative 3D nanomorphology information, we first examine the incident angle dependence of the IP and OOP intensity profiles, as plotted in Fig. 3. The 2D-scattering patterns acquired at 0.15°, 0.30°, 0.45°, and 0.60° are shown in Supplementary Fig. 4. The IP profiles of GISAXS are extracted by the horizontal linecuts at the Yoneda peak positions of each incident angle, whereas the IP profiles of GTSAXS are all performed at **q**_*z*_ = −0.008 Å^−1^. The OOP profiles of GISAXS and GTSAXS are obtained by the vertical linecuts at **q**_*r*_ = 0.006 Å^−1^. Figure 3a, c compare the IP profiles of GISAXS and GTSAXS measured at different incident angles. Obviously, the intensity of GISAXS IP profile is significantly weakened and gradually loses its characteristic variation feature when the incident angle is lifted away from the critical angle. In contrast, the IP profile in the GTSAXS regime exhibits clear structural-related intensity variations, regardless of the incident angle used.

For the OOP profiles, both the GISAXS and GTSAXS show an incident angle dependence mainly due to the effect of reflection caused by the grazing incidence geometry (so-called “geometric distortion”)^21^. Figure 3b, d presents the OOP profiles of GISAXS and GTSAXS measured at different incident angles after the geometric distortion correction^21^. The GISAXS OOP profiles manifest the shift of Yoneda peak with the incident angle, as expected. In contrast, the GTSAXS OOP profiles gradually collapse together when the incident angle increases, indicating that the geometric distortion effect can be ignored at high incident angles (Supplementary Fig. 5). Therefore, it is suggested to perform GTSAXS measurements at a high incident angle, i.e. 0.60^o^, where both the IP and the OOP nanomorphology can be extracted without considering the geometric distortion.

To determine the nanophase separation length scale, simple Born approximation models are readily adapted to fit the scattering intensity profiles. For the P3HT:PC_71_BM system, the hard-sphere model is usually employed^14,31^. The intensity of the scattering can be expressed with the formula:

1 $$I\left({{{{{\bf{q}}}}}}\right)\propto \left\langle {P}_{s}\left({{{{{\bf{q}}}}}},R\right)\right\rangle {S}_{{{{{{\rm{hs}}}}}}}\left({{{{{\bf{q}}}}}},R\right)$$

where $\left\langle {P}_{s}\left({{{{{\bf{q}}}}}},R\right)\right\rangle$ is the form factor of spherical particles with the mean radius *R* under the Schulz distribution^32–34^, ${S}_{{{{{{\rm{hs}}}}}}}\left({{{{{\bf{q}}}}}},R\right)$ is the structure factor with the hard-sphere interparticle interaction calculated under the Percus–Yevick Approximation^35^. Thus, the PC_71_BM domain sizes (2*R*_*g*_) in the IP direction estimated from the GISAXS and GTSAXS IP profiles are 22 nm and 21 nm (solid lines in Fig. 3a, c) respectively, similar to previously reported values^14,31^. It further confirms the capability of GTSAXS in extracting the IP domain sizes as GISAXS does. Here, it is known that multiple scattering features can possibly be observed in GISAXS horizontal linecut profile at the Yoneda peak for P3HT: PCBM blend films, which correspond to the multiple characteristic structures within the thin film, such as the sizes of PCBM aggregates and distances between those aggregates. It is worth noting that practically determined characteristic sizes for nanostructure of P3HT:PCBM system is highly dependent on the specific processing conditions of thin films and can vary within a certain range^36–38^.

Remarkably, the same model could fit well with the GTSAXS OOP profile, giving the PC_71_BM domain size of 5 nm in the OOP direction (Fig. 3d). Here, the excellent feasibility of the simple Born approximation scattering model to fit the OOP profiles of GTSAXS further indicates that the structure probed by GTSAXS should be the vertical domain sizes of phase separation. The relatively smaller acceptor domain size in the OOP direction compared with the domain size in the IP direction is likely owing to the physical confinement imposed in the OOP direction by the thin film structure, which is also observed in NFA-based BHJ films studied below.

### 3D nanomorphology of NF BHJ systems

**Fig. 4:**
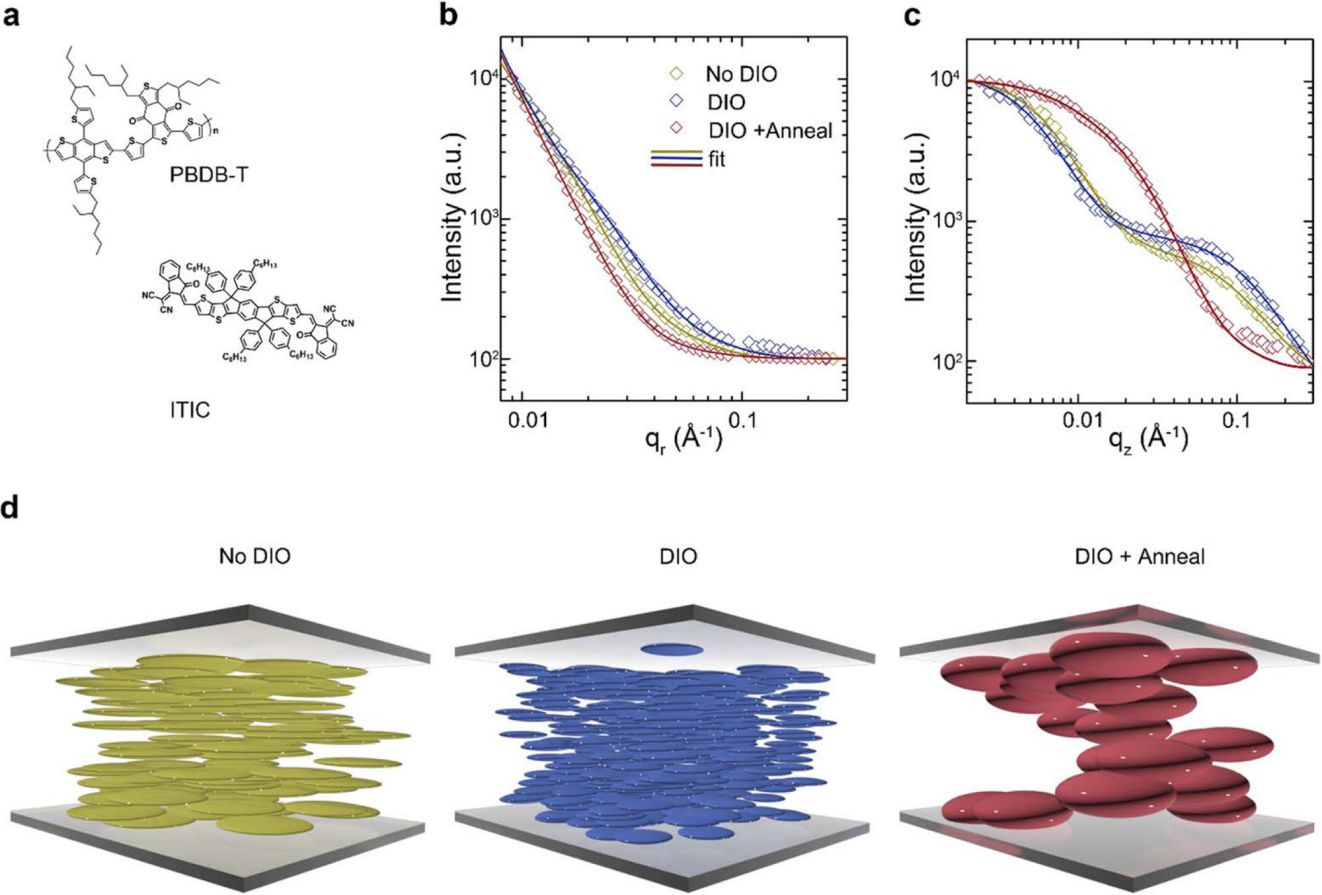
Three-dimensional nanomorphology of non-fullerene-based BHJ. **a** Chemical structures of PBDB-T and ITIC. The GISAXS in-plane **b** and GTSAXS out-of-plane **c** scattering profiles for PBDB-T:ITIC thin films with different fabrication conditions. **d** Schematic showing the three-dimensional domain sizes as colored ellipsoids.

Having demonstrated the capability of GTSAXS in the prototypical fullerene-based system, we now extend this method to NF systems and investigate the influence of OOP nanomorphology on the device performance. We choose a typical NF acceptor-based system, PBDB-T:ITIC^23,24^ (Fig. 4a), and deliberately create a set of diverse morphologies in the active layer by adding additives and thermal annealing. The additive we employ here is DIO, which has been widely applied to optimize the morphology of OPV devices^39–41^. It has been reported that DIO could change the relative solubility and solvent evaporation speed and mediate the degree of phase separation^40^.

Figure 4b, c shows the IP and OOP-scattering profiles of PBDB-T:ITIC blend films, control, with DIO, with DIO, and thermal annealing, respectively. The corresponding 2D-scattering patterns are shown in Supplementary Fig. 6. The line shape and treatment-dependent variation of IP and OOP profiles are evidently distinct. All the IP profiles exhibit a consistent decay in the q region of 0.02–0.05 Å^−1^, corresponding to averaged domain sizes of several tens of nm. In contrast, the OOP profiles of the films without or with DIO resemble each other by a scattering shoulder in the **q** region of ~ 0.1 Å^−1^, suggesting the existence of a much smaller averaged domain size of several nm. Moreover, thermal annealing imposes more prominent changes to the OOP nanomorphology than to the IP nanomorphology. The scattering shoulder of annealed film shifts to a much smaller **q** region (**q** ~ 0.03 Å^−1^), corresponding to a substantial growth of averaged domain size in the OOP direction.

For NF-based system, a theoretical fit of the scattering profile with the fractal-like network model^6,13,42^ as the structure factor in Eq. (1) is capable of estimating pure phase domain sizes, whereas the Debye-Anderson-Brumberger model^43^ is often employed to model the scattering from the amorphous intermixing and diffuse scattering. The fitted IP and OOP domain sizes are summarized in Table 1. Since the scattering of pure PBDB-T film is much weaker than that of pure ITIC film (Supplementary Fig. 7), the fitted pure phase domain sizes are assigned to ITIC. For the pristine PBDB-T:ITIC thin film, the ITIC domain size is found to be 41 nm in the IP direction, similar to previously reported values of such system^41^. In contrast, the OOP domain size is fitted to be 4 nm, almost an order of magnitude smaller than that in the IP direction. The addition of 0.5% DIO gives rise to an ITIC domain shrinking in both IP and OOP direction to 22 nm and 3 nm, respectively. Then, after thermal annealing, the domain size in both directions expanded substantially, to 43 nm in the IP direction and 16 nm in the OOP direction. Figure 4d shows a schematic representation of the ITIC domain inside the active layer, depicted as ellipsoids distributed randomly. All the films present an anisotropic nanomorphology with significant elongation in the IP directions, possibly related to the chemical structure and molecular packing motif of ITIC as well as the anisotropic thin film structure.

**Table 1:** A summary of fitted domain sizes and device characteristics of PBDB-T:ITIC and PM6:Y6 systems.

Blends	Samples	IP domain (nm)	OOP domain (nm)	V_OC_ (V)	*J*_SC_ (mA cm^−2^)	FF (%)	PCE (%)
PBDB-T: ITIC	No DIO	41	4	0.882	16.91	53.7	8.00
	DIO	21	3	0.891	16.20	62.1	8.96
	DIO+Anneal	43	16	0.838	17.01	57.8	8.23
PM6: Y6	Control	13	15	0.871	25.73	65.2	14.60
	CN	12	14	0.876	25.79	66.7	15.08
	CN&annealing	27	28	0.858	26.10	74.5	16.70

**Fig. 5:**
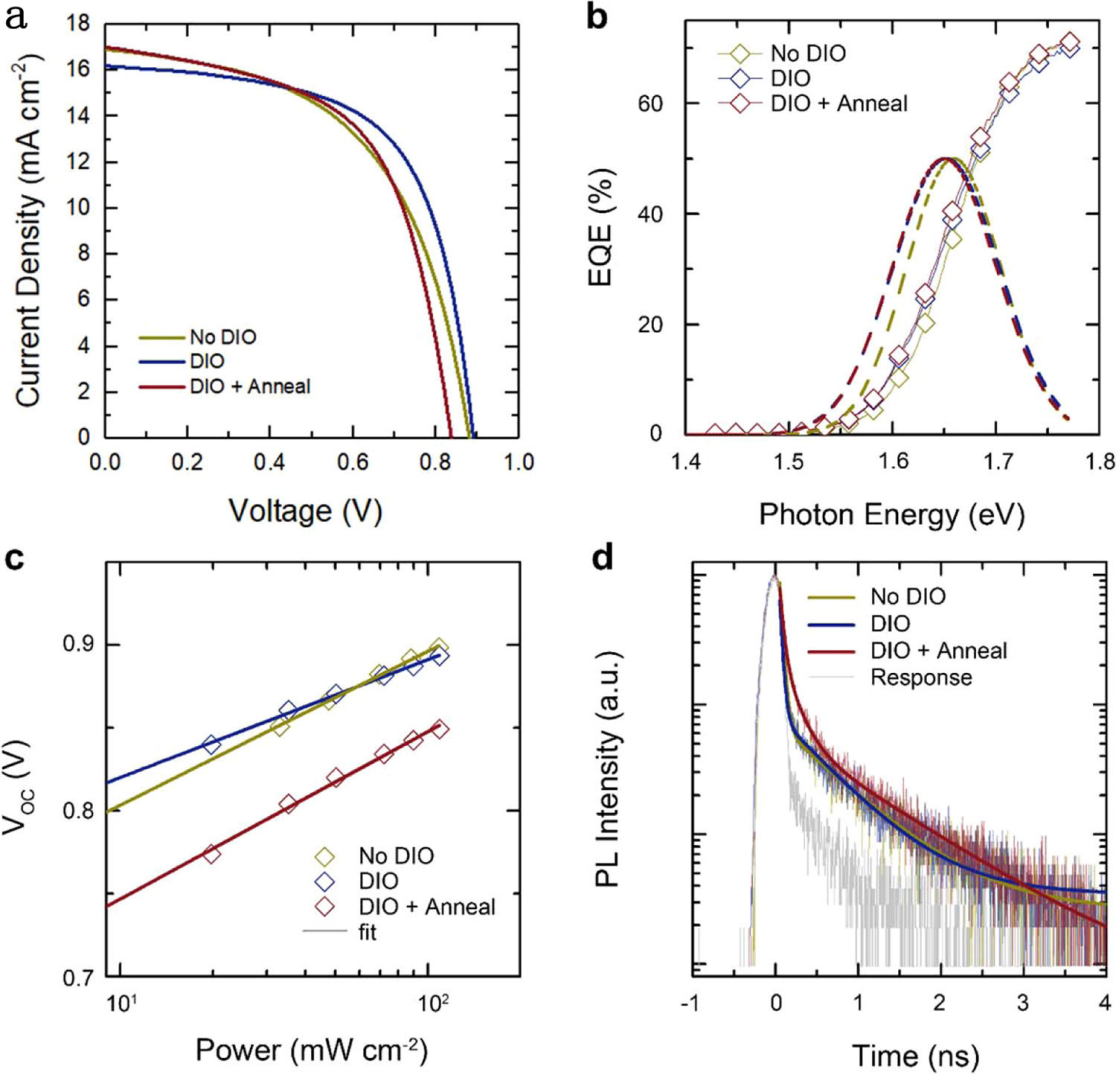
Device characteristics. **a** *J–V* curves of solar cell devices of PBDB-T:ITIC blend as active layer under different fabrication conditions. **b** The EQE edge with respect to photon energy. Dash-lines are the derivatives of the respective spectra. The bandgap of the devices for the control, DIO, and annealed samples, determined from the peak positions of the derivative, are 1.659 eV, 1.652 eV, and 1.650 eV, respectively. **c** Light intensity-dependent *V*_OC_ characteristics of PBDB-T:ITIC devices. **d** Normalized TRPL profiles of the PBDB-T:ITIC excited at 515 nm. The PL signal was collected from 600 to 700 nm and fitted with exponential decay functions.

To understand the influence of 3D nanomorphology, especially the newly obtained OOP domain sizes, on the photovoltaic device performance, solar cells made with PBDB-T:ITIC blend films, control, with DIO, with DIO and thermal annealing, are fabricated. Device parameters are summarized in Table 1 along with the extracted IP and OOP domain sizes for detailed comparison. The *J–V* curves and external quantum efficiency (EQE) are presented in Fig. 5a and Supplementary Fig. 8, respectively. The open-circuit voltage (*V*_OC_) changes from 0.882 V to 0.891 V by adding DIO, then to 0.838 V with further annealing. The corresponding *V*_OC_ loss ($={E}_{g}-q{V}_{{OC}}$) is calculated to be 0.777 eV, 0.761 eV, and 0.812 eV, with the band gap determined from the derivative EQE curve (Fig. 5b). Thus, it is suggested that the DIO & annealing device experiences the most severe recombination, whereas the DIO device has the least. To determine the type of dominant recombination^44,45^, the ideality factor (*n*) is then obtained from the slope of the log-linear plot of *V*_OC_ versus light intensity (Fig. 5c). It is found that *n*_DIO&annealing_ = 1.70 > *n*_control_ = 1.56 > *n*_DIO_ = 1.19. This is consistent with the evolution of 3D nanomorphology. The DIO film has the smallest IP and OOP domain sizes. Consequently, the dominant recombination in the DIO device is bimolecular, owning to the most efficient exciton dissociation. In contrast, the DIO&annealing device has the largest domain size in both IP and OOP directions. Especially, in the OOP direction, the domain size is about five times of the control and DIO samples, which could suppress the exciton dissociation, cause severe monomolecular recombination.

The charge transfer properties of the films are further characterized by time-resolved photoluminescence (TRPL) measurement excited at 515 nm (Fig. 5d). The DIO&annealing sample shows the longest lifetime (0.42 ns), whereas the control sample and the DIO sample decay faster with a lifetime of 0.24 ns. The shorter decay time in the control and DIO samples is related to higher electron transfer efficiency from the donor to the acceptor, directly associated with the observed much smaller OOP domain sizes, which increase the D/A interfaces. Note here, although the control and DIO samples have distinct IP domain sizes, it is not very sensitive to the decay time. It is likely due to the reason that the OOP domain size is significantly smaller than the IP domain size, thus becomes the critical factor here. Therefore, it is confirmed that the newly detected OOP domain size is strongly correlated with the device performance, particularly relevant with the exciton dissociation and charge transfer process. Without the knowledge of OOP domain sizes, it becomes difficult to explain the *V*_OC_ difference between the control and DIO&annealing devices of PBDB-T:ITIC system, as they exhibit very similar IP domain sizes. Besides, the higher FF of DIO & annealing devices can be attributed to the higher crystallinity (Supplementary Fig. 9) and the larger OOP domain size formed connected electron transport pathways.

**Fig. 6:**
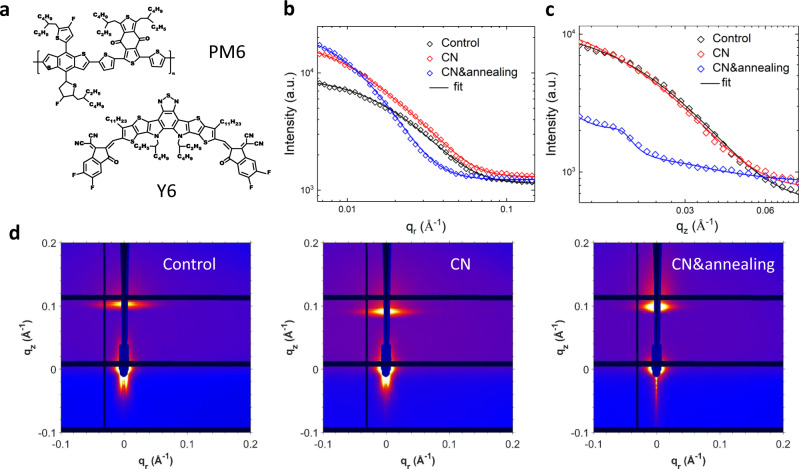
GTSAXS of high-efficiency BHJ system. **a** Chemical structures of PM6 and Y6. **b** The GISAXS in-plane scattering profiles at Yoneda peak and **c** GTSAXS out-of-plane scattering profiles at **q**_*r*_ =  0.006 Å^−1^ for PM6:Y6 blend thin films with different fabrication conditions. **d** Corresponding 2D GI/GTSAXS scattering patterns. The incidence angle is 0.6°.

In order to further demonstrate the generality of the influence of the OOP domain size on the device performance, we also performed GTSAXS measurements on another benchmark system PM6:Y6^25^ (Fig. 6a). The GTSAXS patterns and the corresponding IP and OOP intensity profiles are presented in Fig. 6b, d and Supplementary Fig. 10. The fitted IP and OOP domain sizes and device characteristics are summarized in Table 1 and Supplementary Fig. 11. The atomic force microscopy (AFM) images are shown in Supplementary Fig. 12, presenting similar surface roughness of ~1 nm, consistent with the previous reports^25^. The vertical linecuts at **q**_*r*_ = 0 Å^−1^ below horizon present periodic oscillations, which is very similar to the previously reported results in GISAXS above horizon region^46^. From the periodicity of the fringes (Supplementary Fig. 10a), we have extracted the corresponding characteristic lengths of 79, 80, and 86 nm for the PM6:Y6 thin films, control, with CN, with CN&annealing, respectively. These should most likely originate from the correlation length of thin-film thickness, much larger than the vertical length scale extracted from GTSAXS, suggesting that GTSAXS measures inner domain sizes of phase separation along the surface normal direction. Besides, the vertical intensity profile does not change substantially in the small **q**_*r*_ region, as indicated by the linecuts performed at **q**_*r*_ = 0.006 Å^−1^ and **q**_*r*_ = 0.008 Å^−1^, as shown in Supplementary Fig. 10b, c. This means that the vertical domain size averaged over a large IP length can approximately present the overall averaged vertical domain sizes of the film.

The fitted IP and OOP domain sizes of the pure phase are shown in Table 1. The values are similar to the phase separation length scales (20 ~ 30 nm) indicated in the cross-sectional electron energy loss spectroscopy (EELS) maps of PM6:Y6 (CN&annealing) thin film (Supplementary Fig. 13a), in which the N-rich region refers to the acceptor Y6 phase, whereas the S-rich region refers to the donor enrichment phase. The cross-sectional images show that the domain-like model should also be suitable to extract the quantitative OOP phase structural information for NFA systems, similar to the previously reported PCBM based BHJ thin films^47,48^. In addition, the uniform scattering length density distributions through the whole film thickness extracted from XRR measurements (Supplementary Fig. 14) rule out the possible scattering contribution from enrichment or wetting layer on surface or interface. The domain size changes in response to the additive and thermal annealing are similar for both PBDB-T:ITIC and PM6:Y6 systems: the incorporation of the solvent additive tends to slightly shrink the IP and OOP domain sizes while thermal annealing tends to enlarge them. This correlates with the changing trend of *V*_OC_, which increases slightly first then decreases largely, similar to the trend observed in the PBDB-T:ITIC system. Here, to further eliminate the concern of edge scattering, we have measured the same PM6: Y6 thin film at various cleaved edges, shown in Supplementary Fig. 15. It is evident that both the IP- and OOP-scattering profiles of different edges are similar, and the extracted domain sizes are consistent with each other in a statistical manner. GIWAXS patterns of PM6:Y6 films control, with CN, with CN&annealing (Supplementary Fig. 16) demonstrated that the molecular packing motifs are very similar, whereas the control film has relatively lower crystallinity. Therefore, although the short-circuit current (*J*_SC_) and fill-factor (FF) increase between control and CN added devices may be attributed to the crystallinity increase, the *J*_SC_ and FF increase between CN and CN&annealing devices, which has similar crystallinity, is more likely due to the significant enlargement of vertical domain size, which provides more interconnected charge transport pathways for efficient charge collection. In addition, estimated by the Scherrer equation from the full-width at half-maximum of the—peak position of the PM6:Y6 system (Supplementary Table 1), the size of nano-crystallites within the thin film is ~ 2 nm, much smaller than the length scale (15 ~ 30 nm) extracted from the OOP profile of GTSAXS. Thus, the scattering features on the OOP profile of GTSAXS should not represent the size of the nano-crystallites within domains, but the whole average domain size. It is worth noting that the aspect ratio of IP and OOP domain sizes are much larger than 1 for the PBDB-T:ITIC system and close to 1 for the PM6:Y6 system. This is likely owing to the distinct chemical structure and in turn the packing motif of ITIC and Y6, which is worth further exploring in future work. Furthermore, the IP domain size of the PM6:Y6 system is generally smaller than that of PBDB-T:ITIC, whereas the OOP domain size of PM6:Y6 is larger than that of PBDB-T:ITIC, instead, which is in correspondence with the overall better FF of PM6:Y6, further confirming that the newly revealed OOP domain size of BHJ thin film is closely related to the charge transport efficiency along the vertical direction. Thus, an additional avenue for future device optimization and new photovoltaic material development is allowed with the determination of OOP phase structure for active layer thin film by GTSAXS.

## Discussion

In summary, the application of synchrotron-based GTSAXS in studying the 3D nanomorphology, especially the OOP nanomorphology, of the thin film active layer of organic solar cells has been demonstrated for the first time. It signifies that GTSAXS could offer simultaneous and incident angle-insensitive detection of IP and OOP nanomorphology, while the conventional GISAXS could only provide IP nanomorphology due to the strong reflection and refraction effects. At a relatively higher incidence angle, the GTSAXS profiles can be fitted by simple Born approximation and quantitative domain sizes can be readily extracted. We showed that this technique is applicable to both fullerene systems and NF systems, while different structure models need to be employed for the data analysis (details in Supplementary Information Table 2).

From the device data, the OOP domain size is strongly correlated with the exciton dissociation and charge transfer process, and as a result, can account for the V_OC_ loss. Nevertheless, one still needs to consider other morphological factors, such as crystallinity, crystalline orientation, domain purity and miscibility etc^2–7^, to obtain a comprehensive understanding of the morphology impact on the device performance.

## Methods

### X-ray scattering

GIWAXS and GISAXS measurements were carried out with a Xeuss 2.0 SAXS/WAXS laboratory beamline using a Cu X-ray source (8.05 keV, 1.54 Å) and a Pilatus3R 300 K detector. The incidence angle is 0.2^o^. GTSAXS measurements were performed at beamline BL19U2 of the National Facility for Protein Science Shanghai (NFPS) at Shanghai Synchrotron Radiation Facility (SSRF). The wavelength, *λ*, of X-ray radiation was set as 1.02 Å for P3HT:PC_71_BM samples, 0.918 Å for PBDB-T:ITIC samples, and 1.01 Å for PM6:Y6 samples, respectively. The beam size at sample stage, divergence, and sampling area of the grazing-incident X-ray in this experiment were 400 × 60 μm^2^ (W×H), 80 × 30 μrad (*W*×*H*), and ~ 1.15 mm^2^. Scattered X-ray intensities were collected using a Pilatus 1 M detector (DECTRIS Ltd). The sample-to-detector distance was set such that the detecting range of momentum transfer [***q*** = 4πsinθ/λ, where 2θ is the scattering angle] of SAXS experiments was 0.006–0.47 Å^−1^.

### GTSAXS measurement

To prepare a thin-film sample suitable for GTSAXS characterization, one has to cleave the sample from the center area to create a clean edge (Supplementary Fig. 2) and place the front edge at the center of the goniometer stage to align with the rotational axis of the incidence angle (Supplementary Fig. 1). The sampling region is illustrated in Supplementary Fig. 1b. The theoretical penetration depth of X-ray through silicon substrate versus incident angle at $\lambda =$ 1.014 Å is plotted in Supplementary Fig. 17. The estimated penetration depth is ~ 2.8 μm at an incident angle of 0.6°, which is much thicker than the sample film and thinner than the silicon substrate. Thus, most of the scattering signals should exit from the front edge. Similar to the optical alignment for conventional GIWAXS/GISAXS measurements, we perform one height scan first, which will give a step-wise height profile of the thin film sample, as illustrated in Supplementary Fig. 18a. Then we align the beam to the median height position, followed by a rocking scan of the incident angle. Since the cleaved edge of the sample is at the center of the goniometer stage, the incident angle profile is not symmetric with respect to 0 degree. The intensity decreases when the incident angle moves away from 0 degree on the negative side and remains constant on the positive side (Supplementary Fig. 18b). One can repeat these steps several times to increase the accuracy of the alignment. After the optical alignment, the sample stage is rotated to the desired incident angle for GTSAXS measurements, as shown in Supplementary Fig. 18c. The maximum exposure time for the measurement of the individual samples was set to be 40 s in this synchrotron-based GTSAXS measurement, which can avoid the potential issues of beam damage and simultaneously ensure that enough statistical scattering signals can be collected. Detailed information on model fitting is given in Supplementary Information (Supplementary Table 2).

### GTSAXS sample fabrication

To fabricate P3HT:PC_71_BM thin films, P3HT, and PC_71_BM were simultaneously dissolved in o-DCB to form a solution of concentration 50 mg/ml. The mass ratio of the two components is 1:1. The solution was heated to 55°C for an hour and stirred overnight. To fabricate thin-film samples for X-ray characterization, the solution was spin-coated onto a clean Si substrate. The samples were then annealed for 10 min. To fabricate PBDB-T: ITIC thin films, PBDB-T, and ITIC were dissolved in chlorobenzene. The solution was heated to 55°C for an hour and stirred overnight. For samples with DIO additive, before spin-coating, 0.5 vol% DIO was added and the solution was then stirred for 30 min. The thin films were fabricated by spin-coating solutions on Si substrate. One of the samples was then annealed at 160°C for 10 min. PM6:Y6 thin films were fabricated using solutions with a total concentration of 15.4 mg/ml and D/A ratio of 1:1.2, the spin-coating rate was 3000 rpm. Various PM6:Y6 thin films were fabricated under conditions of as-cast, with 0.5 vol% 1-chloronaphthalene, with 0.5 vol% 1-chloronaphthalene, and thermal annealing at 90°C for 10 min, respectively.

It is worth noting that in the present work, we follow the convention to choose silicon wafer as the substrate for X-ray scattering characterizations^49–51^, because it will impose minimal background scattering signals is compared with ITO substrates and real devices, as shown by the GISAXS linecuts in Supplementary Figs. 19–21. As shown in GIWAXS of thin films on Si substrates and in real devices (Supplementary Figs. 17–18), in these two circumstances, both the IP lamellar peaks and OOP-stacking peaks are highly consistent with each other. Furthermore, the phase structure of thin film on Si substrate is also very similar to that in a real device, as indicated in EELS maps of Supplementary Fig. 13. Thus, the crystalline structure and nanomorphology of NFA-based thin films were not obviously altered when using silicon substrates.

### Device fabrication

The inverted structure was utilized for PBDB-T:ITIC blends as ITO/ZnO/PBDB-T:ITIC/MoO_3_/Al. The ZnO layer was spin-coated then baked at 200 °C in the air for 30 min. PBDB-T:ITIC solutions were prepared and spin-coated on the ZnO layer similarly to GTSAXS samples. After that, the MoO_3_ layer (ca. 6 nm) was thermally evaporated as the hole transporting layer, and the Al (ca. 100 nm) was evaporated as the top electrode.

The conventional structure was utilized for PM6:Y6 blends as ITO/PEDOT: PSS/PM6:Y6/PFN-Br/Ag. The PEDOT:PSS layer was spin-coated then baked at 130 °C in the air for 20 min. PM6:Y6 solutions were prepared and spin-coated on the PEDOT:PSS layer similar to GTSAXS thin film samples. After that, the PFN-Br layer was spin-coated as the electron transporting layer and the Ag (ca. 100 nm) were thermally evaporated as the top electrode.

### Device characterization

The solar cell performance was measured by a Keysight source meter unit under an AM 1.5 G (100 mW cm^−2^) solar simulator using a solar simulator (SS-F5-3A, Enlitech, Taiwan, China). External quantum efficiency data were taken by a solar‐cell spectral‐response measurement system (QER, Enlitech, Taiwan, China).

### TRPL measurement

TRPL of blends was measured using a home-setup microfluorescence system. The excitation light (515 nm) was generated by femtosecond laser (Light Conversion Pharos, 1030 nm, <300 fs, 1 MHz). TRPL decay kinetics were collected using a TCSPC module (PicoHarp 300) and a SPAD detector (IDQ, id100).

### Scanning electron microscopy

A cross-sectional image of a thin-film sample on Si substrate was taken by high-resolution field emission scanning electron microscopy.

### Atomic force microscopy

AFM measurements were obtained by using a Dimension Icon AFM (Bruker) in a taping mode.

### X-ray reflectivity

The XRR measurements were conducted by a Rigaku Smartlab reflectometer with Cu Kα X-ray source (*λ* = 1.541 Å) at China Spallation Neutron Source.

### Cross-sectional sample fabrication

The cross-sectional sample was prepared by a DualBeam FIB-SEM system (Thermo Scientific Scios 2), equipped with platinum (Pt) deposition cartridge and EasyLift nanomanipulator. To minimize the ion beam damage, the sample was protected by a ~ 100 nm carbon coating first by a Sharpie black marker. After that, a several micrometer thick platinum layer was deposited using the gallium ion beam. After rough milling by 30 kV ion beam, the ~ 2 μm thick plate was lifting out and attached on the edge of a copper finger, following thinning processes were using a 5 kV ion beam for minimizing the ion beam damage and the final lamella was <100 nm thick.

### Transmission electron microscopy (TEM) and scanning TEM (STEM)

TEM and STEM of the cross-sectional samples were performed using JEOL JEM-2100F TEM/STEM (Tokyo, Japan) operated at 200 kV. EELS mapping was carried out under 200 kV accelerating voltage with a 13 mrad convergence angle for the optimal probe condition. Energy dispersion of 0.7 eV per channel and 21 mrad collection angle were set up for EELS. High-angle annular dark-field STEM images were acquired with an 89 mrad inner angle simultaneously. The N and S intensity maps were extracted from the EELS mapping by integrating across the energy windows of 401–409 (K edge) and 162–173 (L_2,3_ edge) eV, respectively.

### Reporting summary

Further information on research design is available in the Nature Research Reporting Summary linked to this article.

## Supplementary information

Supplementary Information

Solar Cells Reporting Summary

## Data Availability

The relevant data are available from the authors upon reasonable request.
