# Supplementary material for: Uncovering the out-of-plane nanomorphology of organic photovoltaic bulk heterojunction by GTSAXS
Source: Nat Commun. 2021 Oct 28;12:6226. doi: 10.1038/s41467-021-26510-6 (PMC8553947; doi:10.1038/s41467-021-26510-6)
Supplement: Supplementary file 1 — Supplementary Information [file 41467_2021_26510_MOESM1_ESM.pdf]

Supplementary Information

Uncovering the Out-of-plane Nanomorphology of Organic Photovoltaic Bulk Heterojunction by GTSAXS

*Xinxin Xia[1, 11], Tsz-Ki Lau[1, 11], Xuyun Guo[2], Yuhao Li[1], Minchao Qin[1], Kuan Liu[3], Zeng Chen[4], Xiaozhi Zhan[5,6], Yiqun Xiao[1], Pok Fung Chan[1], Heng Liu[1], Luhang Xu[1], Guilong Cai[1], Na Li[7], Haiming Zhu[4], Gang Li[3], Ye Zhu[2], Tao Zhu[8], Xiaowei Zhan[9], Xun-Li Wang[10] and Xinhui Lu[1]**

[1] Department of Physics, The Chinese University of Hong Kong, New Territories, Hong Kong, 999077, China.

[2] Department of Applied Physics, Research Institute for Smart Energy, The Hong Kong Polytechnic University, Hung Hom, Hong Kong, China

[3] Department of Electronic and Information Engineering, Research Institute for Smart Energy (RISE), The Hong Kong Polytechnic University, Hung Hom, Kowloon, Hong Kong, China

[4] Center for Chemistry of High-Performance & Novel Materials, Department of Chemistry, Zhejiang University, Hangzhou 310027, Zhejiang, China

[5] Spallation Neutron Source Science Center, Dongguan 523803, China

[6] Institute of High Energy Physics, Chinese Academy of Sciences, Beijing 100049, China

[7] National Facility for Protein Science in Shanghai, Zhangjiang Laboratory, Shanghai Advanced Research Institute, Chinese Academy of Science, No.333, Haike Road, Shanghai, 201204, People's Republic of China

[8] Beijing National Laboratory for Condensed Matter Physics and Institute of Physics, Chinese Academy of Sciences, Beijing 100190, China

[9] School of Materials Science and Engineering, Peking University, Beijing 100871, China.

[10] Department of Physics and Center for Neutron Scattering, City University of Hong Kong, Kowloon, Hong Kong, China

[11] These authors contribute equally to this work: Xinxin Xia, Tsz-Ki Lau.

*Email: xinhui.lu@cuhk.edu.hk (X. L.)

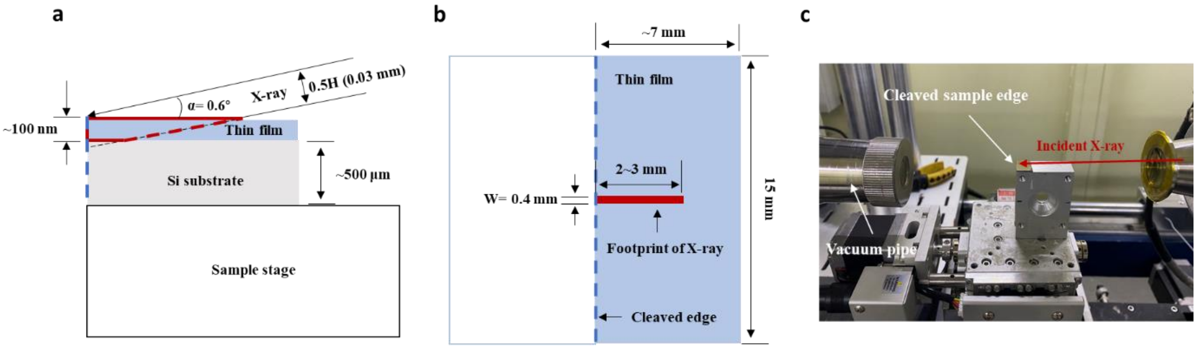

Supplementary Fig. 1 Schematic cartoons of GTSAXS setup geometry: **a** side view, **b** top view of sample stage. **c** Photograph of sample stage setup for GTSAXS.

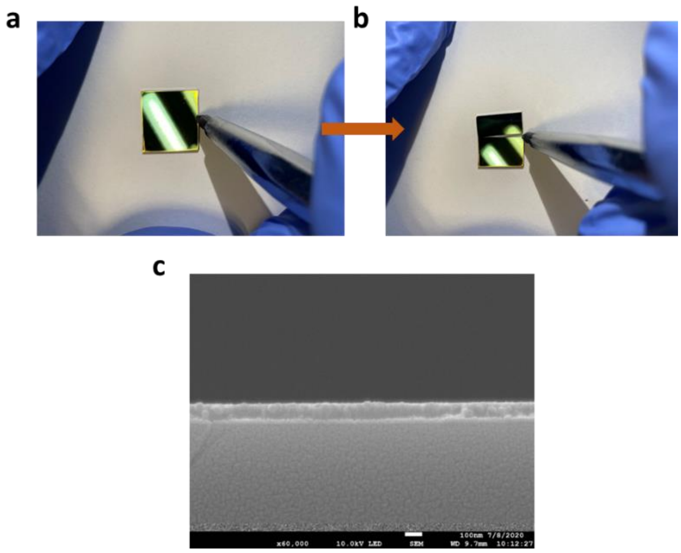

Supplementary Fig. 2 a, b Photographs of the process to cleave thin film sample. **c** Cross-sectional SEM image of the cleaved PBDB-T:ITIC thin film on Si substrate for GTSAXS measurement.

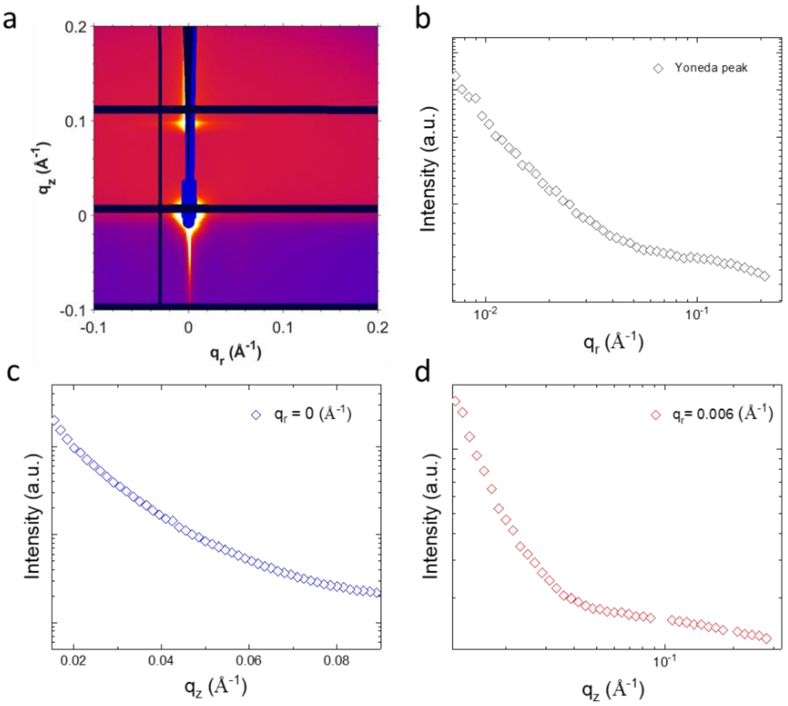

Supplementary Fig. 3 a 2D GISAXS/GTSAXS scattering pattern of bare silicon wafer at 0.6° incidence. **b** Horizontal line-cut at Yoneda peak and vertical line-cuts at **c** $\mathbf{q}_r$ = 0 Å^{-1} and **d** $\mathbf{q}_r$ = 0.006 Å^{-1}, respectively.

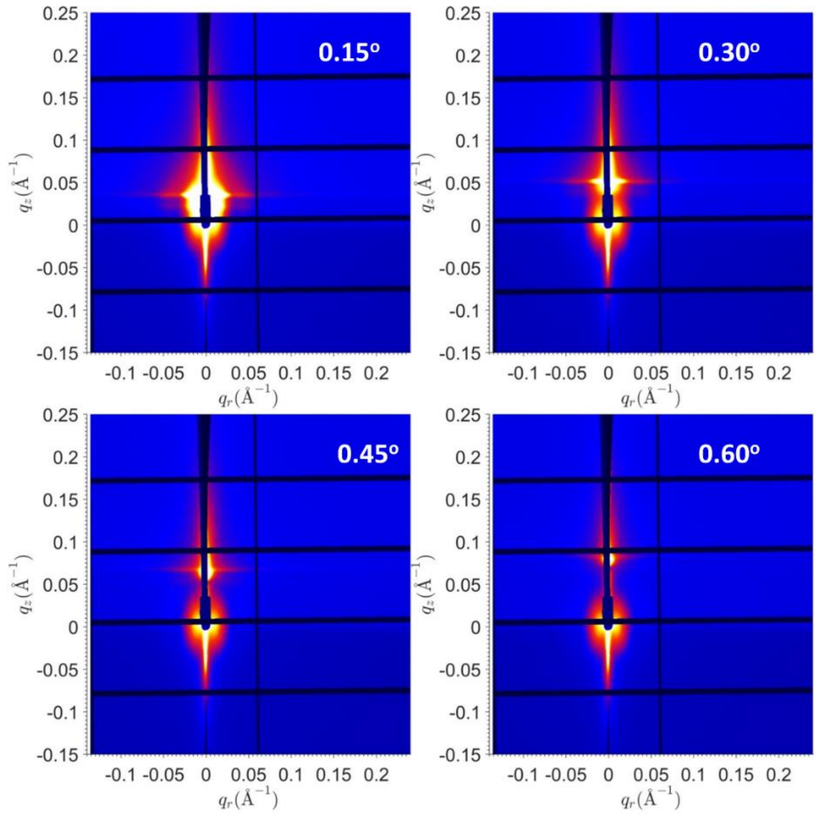

Supplementary Fig. 4 2D scattering patterns of P3HT:PC_{71}BM thin films measured at different incident angles of 0.15°, 0.30°, 0.45° and 0.60°.

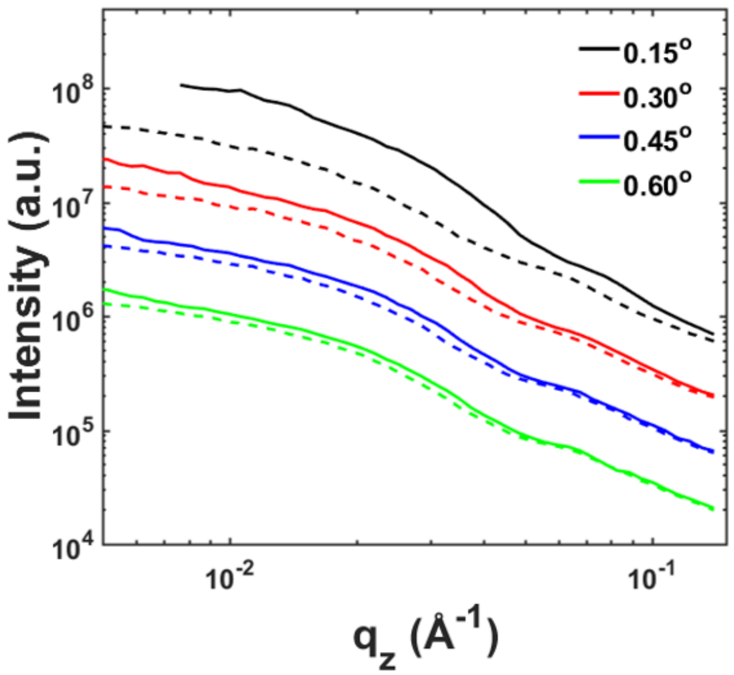

Supplementary Fig. 5 GTSAXS OOP intensity profile (dashed lines) before and (solid lines) after correction for refraction obtained from different incident angles.

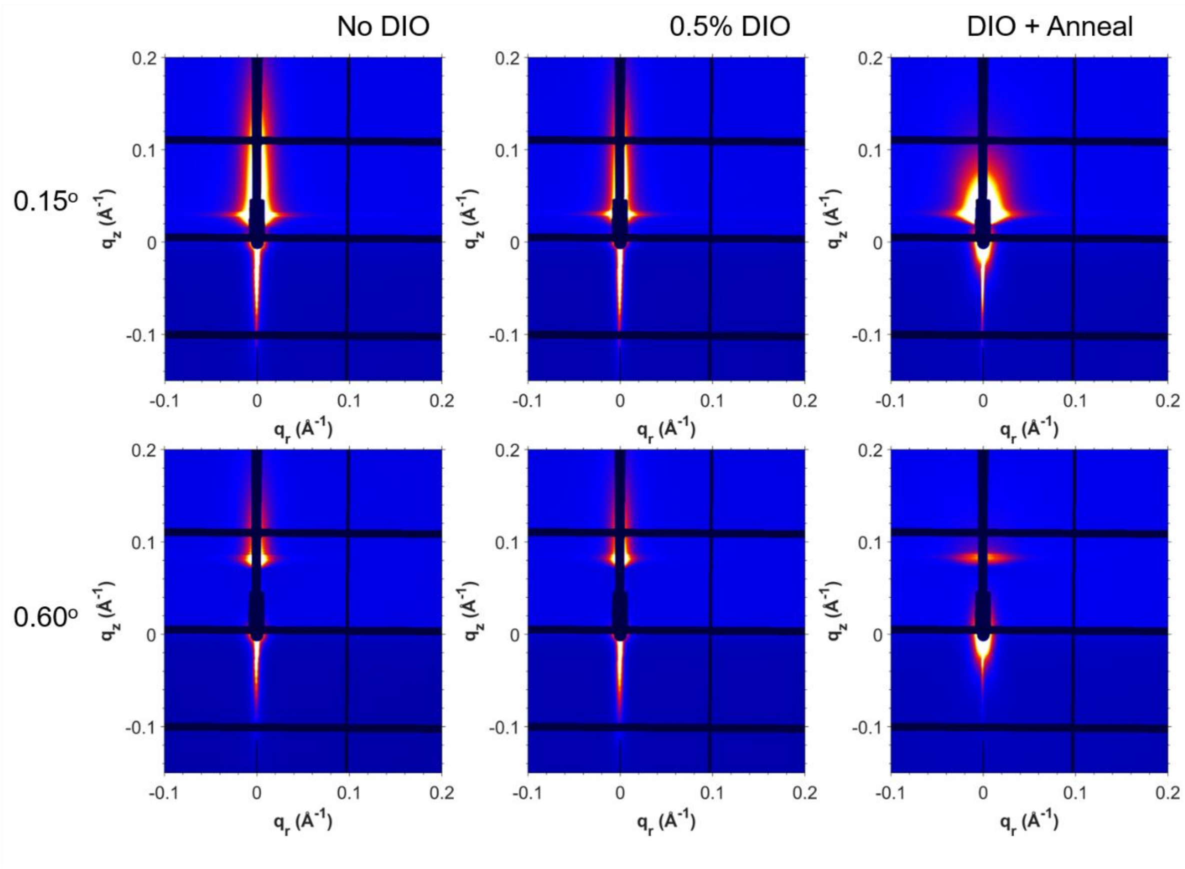

Supplementary Fig. 6 2D GISAXS/GTSAXS scattering patterns of PBDB-T:ITIC thin films under different conditions measured at 0.15° and 0.60° incidence.

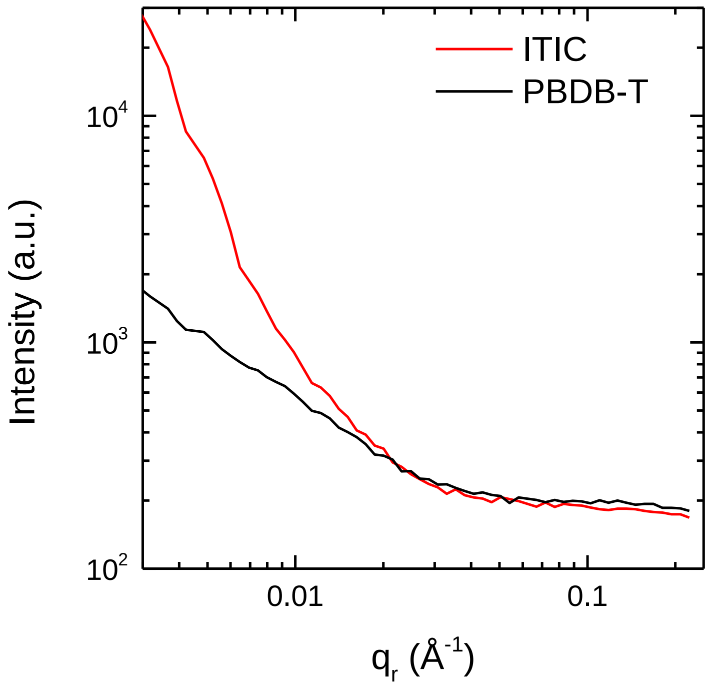

Supplementary Fig. 7 The IP GISAXS scattering profile of pure ITIC and PBDB-T thin films.

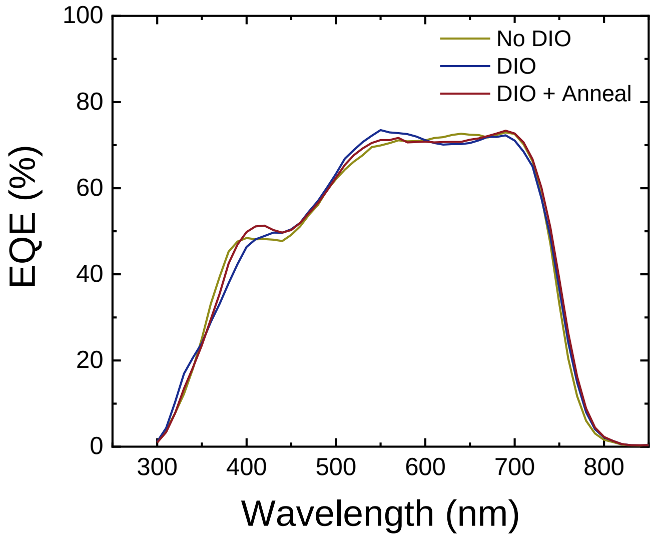

Supplementary Fig. 8 EQE spectra of PBDB-T:ITIC device under different fabrication conditions.

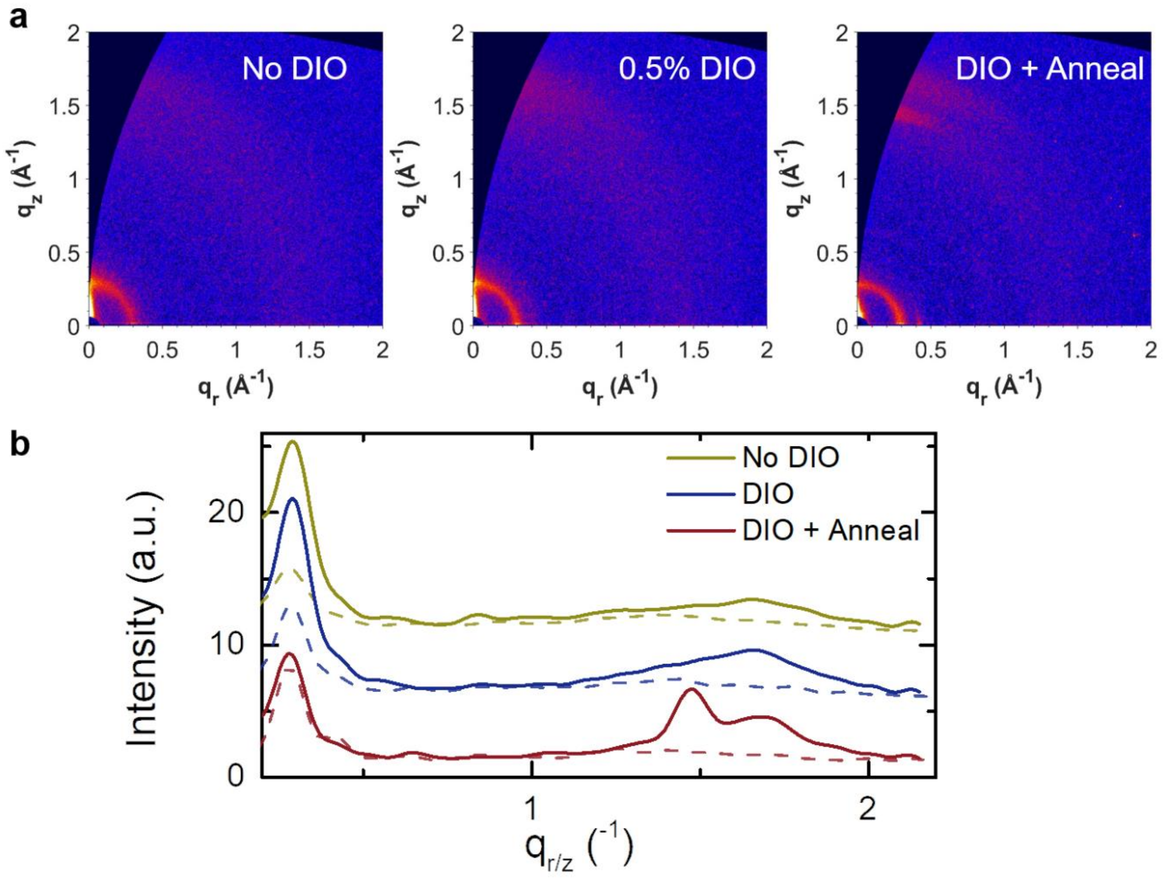

Supplementary Fig. 9 a 2D GIWAXS patterns of PBDB-T:ITIC thin films. **b** the corresponding intensity profile in the (solid lines) out-of-plane and (dashed lines) in-plane directions.

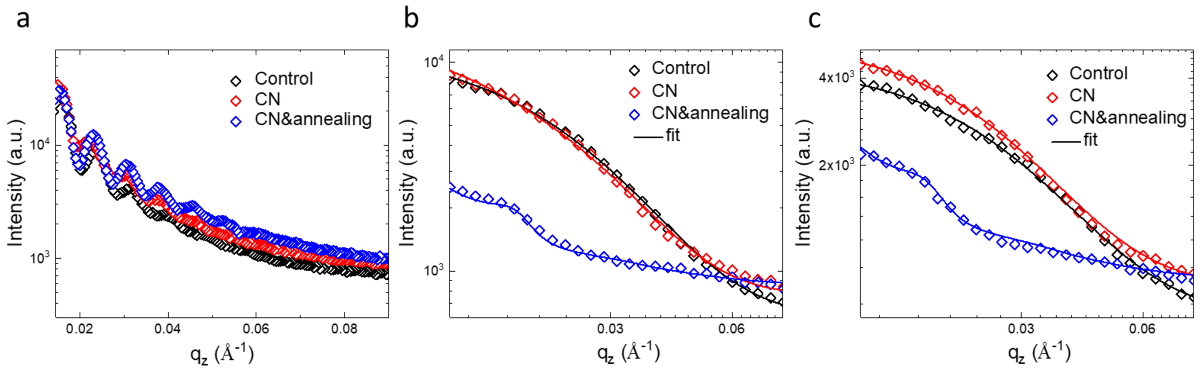

Supplementary Fig. 10 GTSAXS out-of-plane scattering profiles at **a** $\mathbf{q}_r$ = 0 Å^{-1}, **b** $\mathbf{q}_r$ = 0.006 Å^{-1} and **c** $\mathbf{q}_r$ = 0.008 Å^{-1} respectively for PM6:Y6 blend thin films with different fabrication conditions.

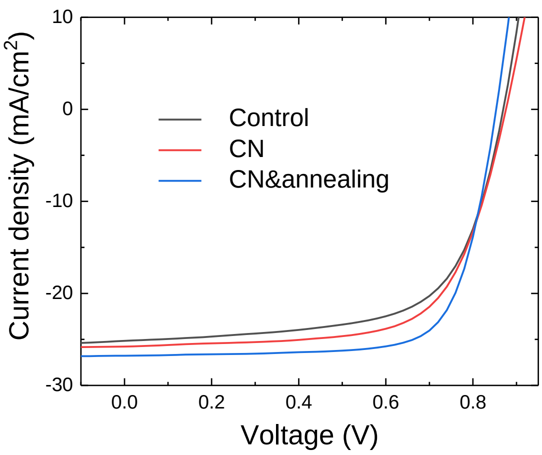

Supplementary Fig. 11 *J*-*V* curves of solar cell devices with PM6:Y6 blend as active layer under different fabrication conditions.

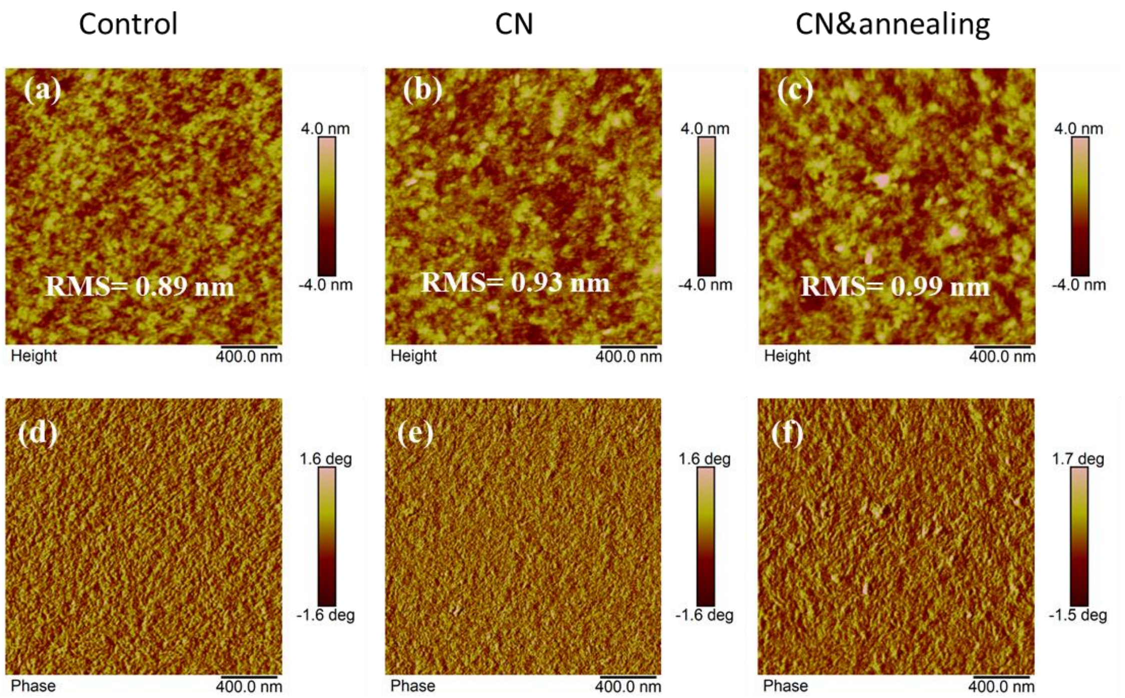

Supplementary Fig. 12 AFM **a-c** height images and **d-f** phase images of PM6:Y6 thin films processed under various conditions.

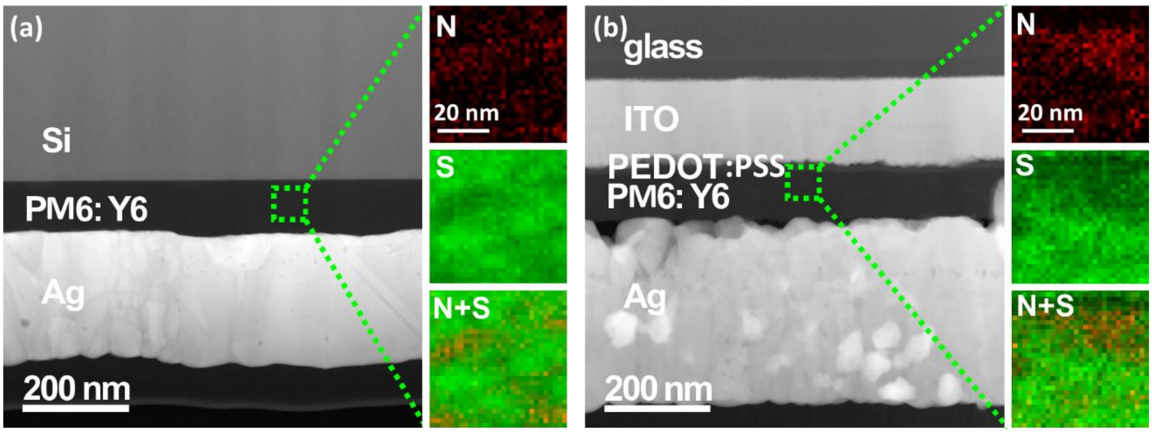

Supplementary Fig. 13 Cross-sectional HAADF-STEM images and corresponding EELS maps of PM6: Y6 (CN&annealing) thin films **a** on a Si substrate and **b** in a real device.

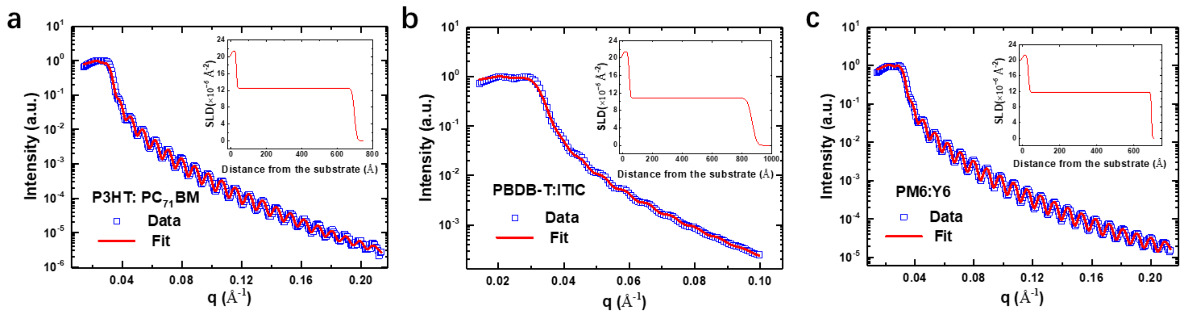

Supplementary Fig. 14 The XRR data and fits for **a** P3HT: PC71BM, **b** PBDB-T: ITIC (as cast) and **c** PM6: Y6 (as cast). The insets show the SLD profile along the vertical direction for each BHJ thin film.

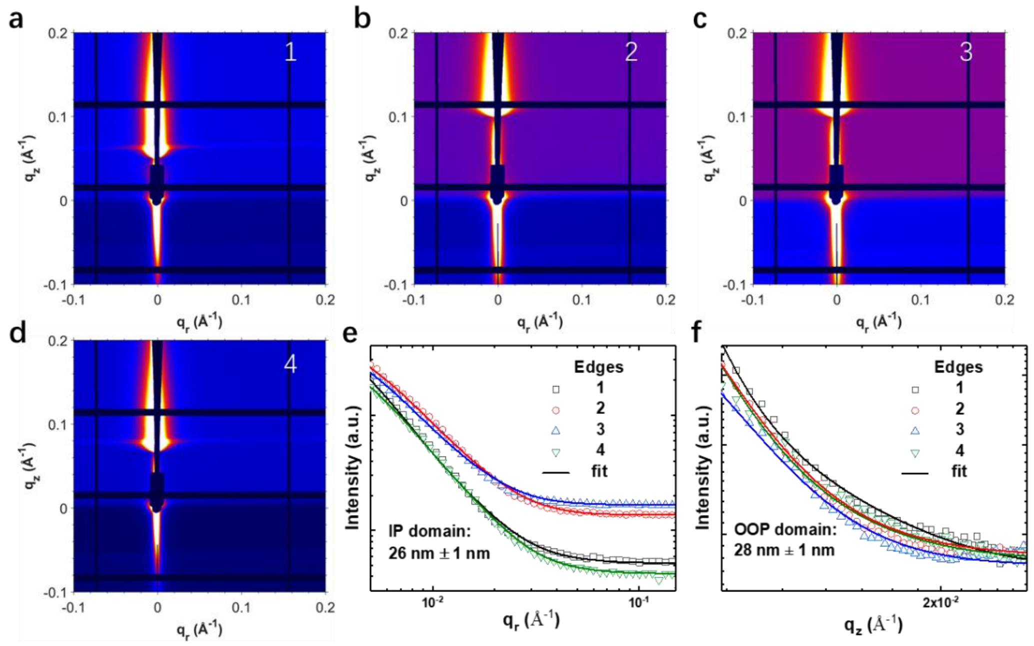

Supplementary Fig. 15 GTSAXS **a-d** patterns and **e-f** corresponding linecuts of PM6: Y6 thin film (CN&annealing) measured at various cleaved edges of sample on Si substrate. Note here that larger incident angle was applied to edge 2 and 3 deliberately to verify the influence of different incident angles.

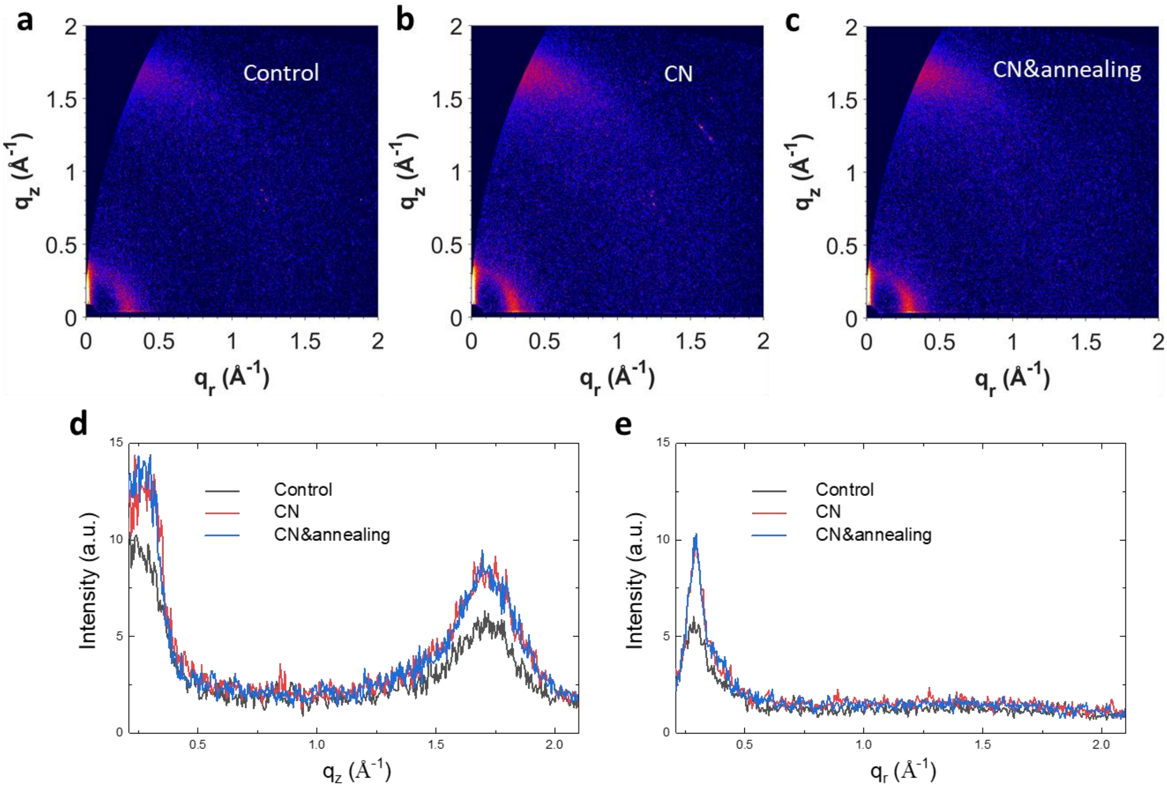

Supplementary Fig. 16 a-c 2D GIWAXS patterns and **d, e** corresponding 1D linecuts of PM6:Y6 thin films processed under different conditions.

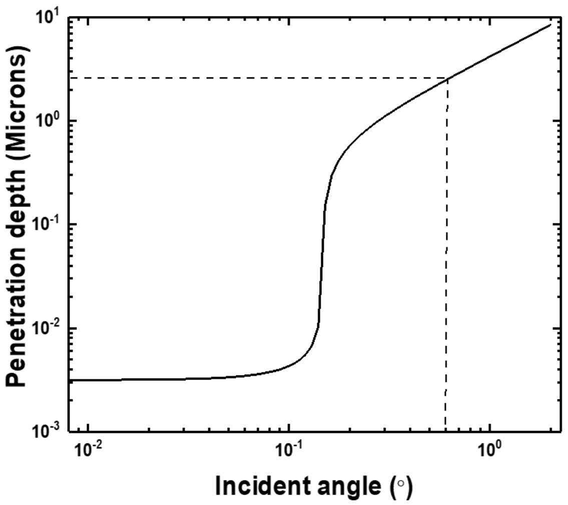

Supplementary Fig. 17 The estimated penetration depth of X-ray through silicon substrate versus incident angle.

The penetration depth of X-ray beam through silicon substrate is estimated by the following equations:

$$\tau(\alpha) = \frac{\sqrt{2}\lambda}{4\pi}\left[\sqrt{(\alpha^2 - {\alpha_c}^2)^2 + 4\beta^2} - (\alpha^2 - {\alpha_c}^2)\right]^{-1/2} \quad (1)$$

$$\alpha_c = \sqrt{2\delta} \quad (2)$$

$$\delta = \frac{n_a r_e \lambda^2}{2\pi} f_1 \quad (3)$$

$$\beta = \frac{n_a r_e \lambda^2}{2\pi} f_2 \quad (4)$$

where $\tau(\alpha)$ is the penetration depth of X-ray under incident angle α, λ is the wavelength of X-ray, α_c is the critical angle of the silicon substrate related to the X-ray wavelength, δ is the real part that describes the dispersive aspect of the wave-matter interaction, β is the imaginary part that is related to the absorption coefficient, r_e is the classical electron radius of 2.818×10^{-15} m, and the atomic scattering factors (f_1 and f_2) of silicon element can be obtained from the website 'https://henke.lbl.gov/optical_constants/asf.html'. The number density n_a is calculated by the equation of $n_a = \rho N_a / M_a$, where ρ is the physical density of silicon (2.33 g/cm^3 for single crystal silicon), N_a is the Avogadro constant of 6.022×10^{23} mol^{-1} , and M_a is molar

mass of silicon. Hence, theoretical penetration depth of X-ray through silicon substrate *versus* incident angle at $\lambda = 1.014$ Å is plotted in Supplementary Fig. 17. The estimated penetration depth is about 2.8 μm at an incident angle of 0.6°, which is much thicker than the sample film and thinner than the silicon substrate.

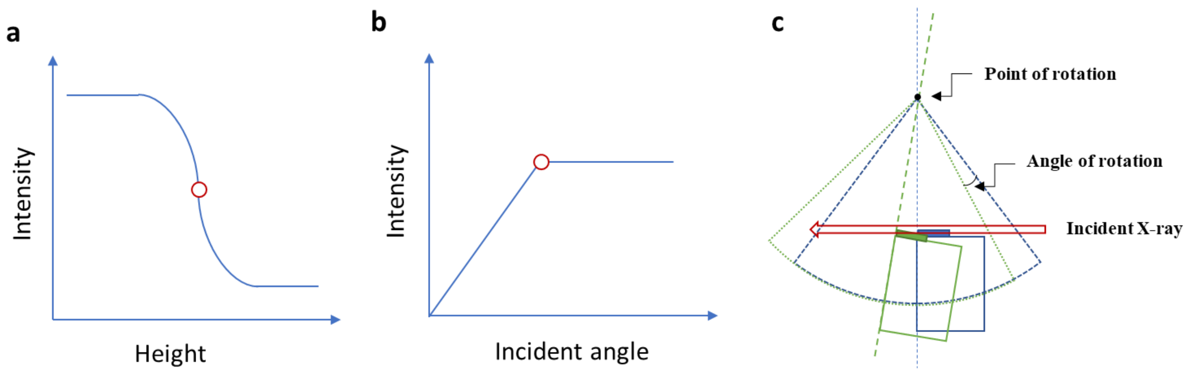

Supplementary Fig. 18 Schematic of **a** height, **b** incident angle scan plots for optical alignment of GTSAXS measurement and **c** rotation of sample stage. The red circles in **a** and **b** refer to where the sample stage is set to after corresponding scanning.

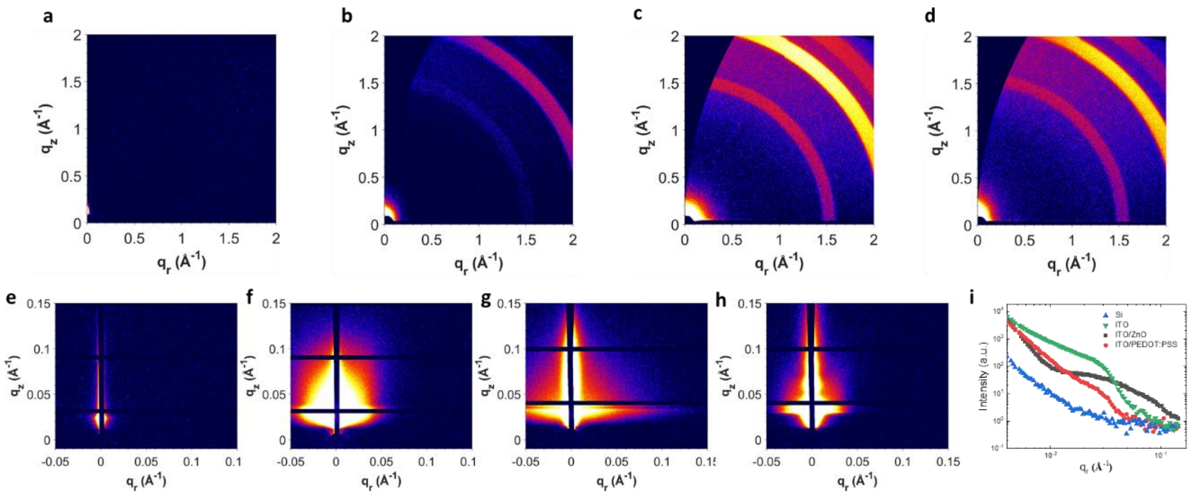

Supplementary Fig. 19 2D GIWAXS and GISAXS patterns of different substrates: **a, e** Si wafer, **b, f** ITO glass, **c, g** ITO glass coated with ZnO and **d, h** ITO glass coated with PEDOT: PSS. **i** $\mathbf{q}_r$ - linecuts at Yoneda peak of GISAXS. The incidence angle is 0.2°.

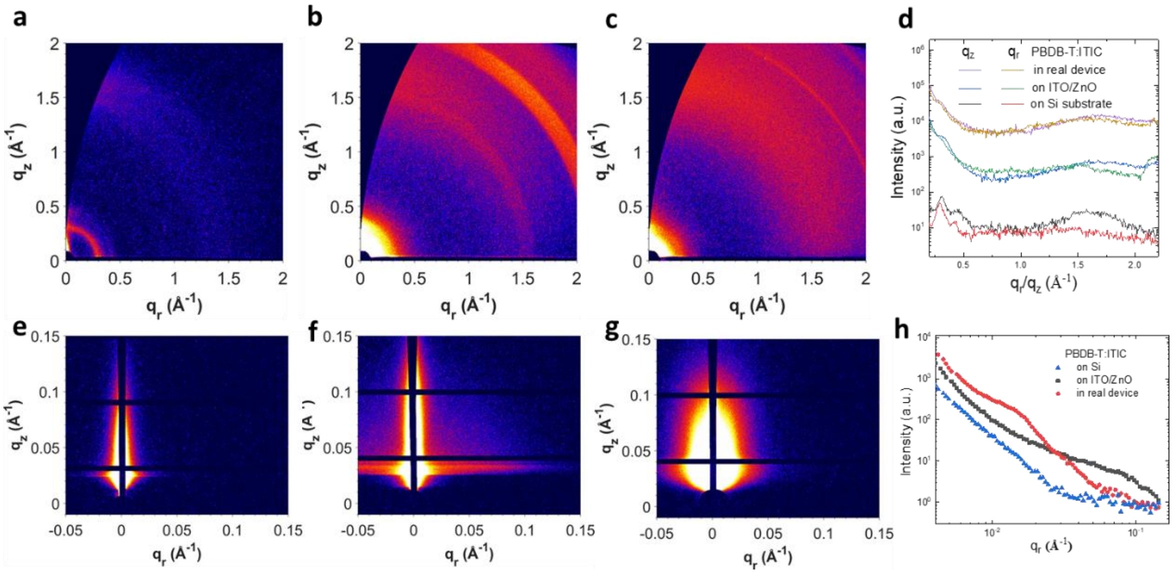

Supplementary Fig. 20 2D GIWAXS and GISAXS patterns of PBDB-T: ITIC thin films **a, e** on Si wafer, **b, f** on ITO glass coated with ZnO, and **c, g** in real devices, respectively. **d** Corresponding 1D GIWAXS linecut profiles, **h** $\mathbf{q}_r$ - linecuts at Yoneda peak of GISAXS. The incidence angle is 0.2°.

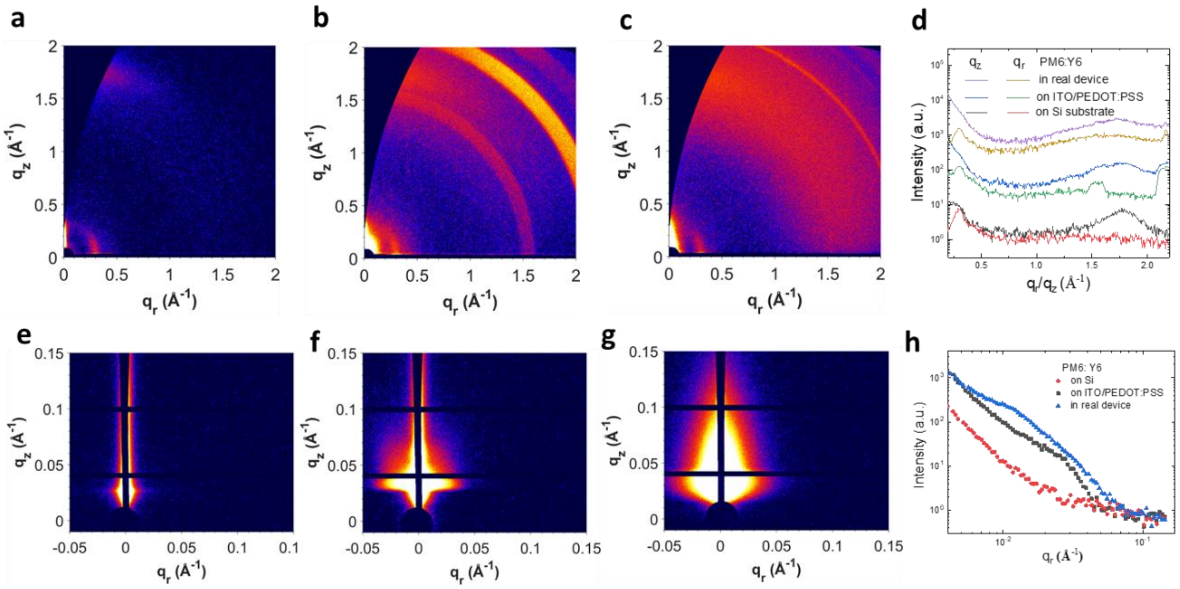

Supplementary Fig. 21 2D GIWAXS and GISAXS patterns of PM6:Y6 thin films **a, e** on Si wafer, **b, f** on ITO glass coated with PEDOT: PSS, and **c, g** in real devices, respectively. **d** Corresponding 1D GIWAXS linecut profiles, **h** $\mathbf{q}_r$ - linecuts at Yoneda peak of GISAXS. The incidence angle is 0.2°.

Supplementary Table 1 Parameters extracted from GIWAXS of PM6:Y6 blend films, related to **Supplementary Fig. 16**.

Samples	Control	CN	CN&annealing
Peak positions of π-π stacking ($Å^{-1}$)	1.70	1.70	1.71
π-π stacking distance (Å)	3.70	3.70	3.67
FWHM of π-π stacking peak ($Å^{-1}$)	0.311	0.323	0.293
Coherence length of π-π stacking (Å)	18.8	18.1	19.9

Supplementary Table 2 Summary of parameters in the scattering models used to fit the IP and OOP profiles of GTSAXS.

Blends	Samples	IP/OOP	ξ_{DAB} /Å	Hard sphere model			Fractal-like network model				2Rg /nm
				R /Å	Z	ϕ /%	R /Å	Z	D	ξ /Å	
$P3HT:PC_{71}BM$	Annealed	IP	200	139	44	20.0	-	-	-	-	22
		OOP	294	35	2	25.3	-	-	-	-	5
PBDB-T:ITIC	No DIO	IP	59	-	-	-	26	16	3	83	41
		OOP	123	-	-	-	4	10	3	7	4
	DIO	IP	105	-	-	-	5	10	3	43	21
		OOP	144	-	-	-	5	5	3	6	3
	DIO+ anneal	IP	350	-	-	-	20	9	3	89	43
		OOP	100	-	-	-	15	10	3	33	16
PM6:Y6	Control	IP	60	-	-	-	11	4	3	27	13
		OOP	60	-	-	-	25	5	3	30	15
	CN	IP	74	-	-	-	15	5	3	26	12
		OOP	90	-	-	-	29	5	3	29	14
	CN& annealing	IP	83	-	-	-	30	6	3	56	27
		OOP	320	180	1	33.9	-	-	-	-	28

Supplementary Note 1

The scattering intensity is modeled as

$$I(\mathbf{q}) = A1\langle P(\mathbf{q}, R, z)\rangle S(\mathbf{q}, R) + A2\ DAB(\mathbf{q}) + A3$$

Here, A1, A2, A3 are prefactors, the background signal is modeled as $DAB(\mathbf{q})$ with DAB model (*J. Appl. Phys.* **1957**, 28, 679-683):

$$DAB(\mathbf{q}) = \frac{8\pi\xi_{DAB}^3}{(1+\mathbf{q}^2\xi_{DAB}^2)^2}$$

where ξ_{DAB} is the correlation length of amorphous intermixing phases. $\langle P(\mathbf{q},R,z)\rangle$ is the averaged form factor. Here, we employ the spherical form factor $P(\mathbf{q},r)$ following Schulz distribution $f(r,R,z)$ with the mean radius R and radius distribution $z > -1$ (*Phys. Rev. A*, **1987**, 35, 2200):

$$P(\mathbf{q},r) = \left[\frac{3(\sin\mathbf{q}r - \mathbf{q}r\cos\mathbf{q}r)}{(\mathbf{q}r)^3}\right]^2$$

$$f(r,R,z) = \left(\frac{z+1}{R}\right)^{z+1} r^z \exp\left[-\left(\frac{z+1}{R}r\right)\right]\frac{1}{\Gamma(z+1)}$$

$$\langle P(\mathbf{q},R,z)\rangle = \int_0^\infty P(\mathbf{q},r)f(r,R,z)dr$$

where $\Gamma(z+1)$ is the Gamma function. $S(\mathbf{q},R)$ is the structure factor which may differ for different systems. For the P3HT:$PC_{71}BM$ system, we adopted a polydisperse hard sphere structure factor under Percus–Yevick Approximation (*Phys. Rep.* **1996**, 272, 216-372):

$$x = 1 - 12\phi\left(\frac{(1+0.5\phi)(-1+\mathrm{Cos}[2\mathbf{q}R])}{4\mathbf{q}^2R^2(1-\phi)^2} + \frac{(1+2\phi)(2\mathbf{q}R - \mathrm{Sin}[2\mathbf{q}R])}{8\mathbf{q}^3R^3(1-\phi)^2}\right)$$

$$y = 12\phi\left(\frac{(1+2\phi)(\frac{1}{4\mathbf{q}R} + \frac{-1+\mathrm{Cos}[2\mathbf{q}R]}{8\mathbf{q}^3R^3})}{(1-\phi)^2} - \frac{(1+0.5\phi)(2\mathbf{q}R - \mathrm{Sin}[2\mathbf{q}R])}{4\mathbf{q}^2R^2(1-\phi)^2}\right)$$

$$S_{hs}(q,R,\phi) = \frac{1}{x^2+y^2}$$

where R is the mean radius of spheres and ϕ is volume fraction of hard spheres. The domain size of pure phase is determined by $2Rg$, where the gyration radius $Rg = [3R^2/5]^{1/2}$.

For the PBDB-T:ITIC and PM6:Y6 systems, we mainly used a fractal-like network structure factor (*Energy Environ. Sci.* **2013**, 6, 1938-1948):

$$S_f(\mathbf{q}, R, \xi) = 1 + \frac{\sin[(D-1)\tan^{-1}(\mathbf{q}\xi)]}{(\mathbf{q}R)^D} \frac{D\Gamma(D-1)}{[1+1/(\mathbf{q}\xi)^2]^{(D-1)/2}}$$

where ξ is the correlation length of the fractal-like network formed by the aggregation of primary particles with the mean radius *R*. Here, the primary particles correspond to small molecular crystallites and the correlation length corresponds to the pure phase domain. The fractal dimension *D* is fixed to 3 here. The domain size of pure phase is determined by $2Rg$, where $Rg = [D(1+D)/2]^{1/2}\ \xi$ is the gyration radius of this fractal-like network. Note here for the PM6:Y6 CN&annealing sample, hard sphere model was employed, because it is more suitable than the fractal-like network model to fit the sharp shoulder-like scattering feature of the OOP profile.
