# Supplementary material for: Uncovering the out-of-plane nanomorphology of organic photovoltaic bulk heterojunction by GTSAXS
Source: Nat Commun. 2021 Oct 28;12:6226. doi: 10.1038/s41467-021-26510-6 (PMC8553947; doi:10.1038/s41467-021-26510-6)
Supplement: Supplementary file 2 — Solar Cells Reporting Summary [file 41467_2021_26510_MOESM2_ESM.pdf]

nature research

Corresponding author(s): Xinhui Lu

Solar Cells Reporting Summary

Nature Research wishes to improve the reproducibility of the work that we publish. This form is intended for publication with all accepted papers reporting the characterization of photovoltaic devices and provides structure for consistency and transparency in reporting. Some list items might not apply to an individual manuscript, but all fields must be completed for clarity.

For further information on Nature Research policies, including our data availability policy, see Authors & Referees.

Experimental design

Please check: are the following details reported in the manuscript?

1. Dimensions

Area of the tested solar cells

- [x] Yes — The area of the tested solar cells is 3.8 mm2
- [ ] No — *Explain why this information is not reported/not relevant.*

Method used to determine the device area

- [x] Yes — The device area is defined by the overlaping area of ITO and Ag grids, the widths of ITO and Ag grids are 2 and 1.9 mm, respectively.
- [ ] No — *Explain why this information is not reported/not relevant.*

2. Current-voltage characterization

Current density-voltage (J-V) plots in both forward and backward direction

- [ ] Yes — *State where this information can be found in the text.*
- [x] No — Generally, Organic solar cells don't show the hysteresis between forward and backward J-V scan, and we only scanned the devices in forward direction.

Voltage scan conditions
For instance: scan direction, speed, dwell times

- [x] Yes — The scan direction is -0.2 V to 1.2 V, with a scan step of 0.01 V and dwell time is 1 ms.
- [ ] No — *Explain why this information is not reported/not relevant.*

Test environment
For instance: characterization temperature, in air or in glove box

- [x] Yes — Devices were tested at room temperature in N2-filled glove box.
- [ ] No — *Explain why this information is not reported/not relevant.*

Protocol for preconditioning of the device before its characterization

- [ ] Yes — *State where this information can be found in the text.*
- [x] No — No preconditioning protocol is used as the performance of organic solar cell exhibits no preconditioning dependence.

Stability of the J-V characteristic
Verified with time evolution of the maximum power point or with the photocurrent at maximum power point; see ref. 7 for details.

- [ ] Yes — *State where this information can be found in the text.*
- [x] No — We have not included stability of the J-V characteristic in the manuscript as our study focuses on the revealing of OOP nanomorphology for OPV BHJ and structure-performance relationship.

3. Hysteresis or any other unusual behaviour

Description of the unusual behaviour observed during the characterization

- [ ] Yes — *State where this information can be found in the text.*
- [x] No — We did not observe hysteresis or any other unusual behaviour during the characterization of our organic solar cells.

Related experimental data

- [ ] Yes — *State where this information can be found in the text.*
- [x] No — No unusual behaviour was observed.

4. Efficiency

External quantum efficiency (EQE) or incident photons to current efficiency (IPCE)

- [x] Yes — EQE is provided as Supplementary Fig. 8 in the Supplementary Information.
- [ ] No

A comparison between the integrated response under the standard reference spectrum and the response measure under the simulator

- [ ] Yes — These are not relevant to the main focus of this study.
- [x] No

For tandem solar cells, the bias illumination and bias voltage used for each subcell

- [ ] Yes — *State where this information can be found in the text.*
- [x] No — We do not report tandem soalr cells in our work.

5. Calibration

Light source and reference cell or sensor used for the characterization

☒ Yes ☐ No

The light source is a solar simulator (SS-F5-3A, Enlitech). The light intensity is calibrated with a monocrystalline silicon reference cell (purchased from Enli Tech. Co., Ltd., Taiwan).

Confirmation that the reference cell was calibrated and certified

☒ Yes ☐ No

The standard monocrystalline silicon reference cell with KG2 filter was purchased from Enli Tech. Co., Ltd., Taiwan and was calibrated and certified.

Calculation of spectral mismatch between the reference cell and the devices under test

☐ Yes ☒ No

State where this information can be found in the text.

We did not calculate the mismatch between the reference cell and the devices under test, for this value is very small with 3A solar simulator after calibrating reference cell with KG-2 filter.

6. Mask/aperture

Size of the mask/aperture used during testing

☐ Yes ☒ No

We did not use mask or aperture during testing.

Variation of the measured short-circuit current density with the mask/aperture area

☐ Yes ☒ No

These measurements have not been performed as we did not aim to report the record-efficiency.

7. Performance certification

Identity of the independent certification laboratory that confirmed the photovoltaic performance

☐ Yes ☒ No

The photovoltaic performance of our organic solar cells has not been confirmed from the independent certification laboratory, as we did not aim to report the record-efficiency.

A copy of any certificate(s)
Provide in Supplementary Information

☐ Yes ☒ No

Not applicable.

8. Statistics

Number of solar cells tested

☒ Yes ☐ No

At least 10 independent devices were tested for each material system.

Statistical analysis of the device performance

☒ Yes ☐ No

We have included the average device characteristics in Table 1.

9. Long-term stability analysis

Type of analysis, bias conditions and environmental conditions
For instance: illumination type, temperature, atmosphere humidity, encapsulation method, preconditioning temperature

☐ Yes ☒ No

We did not include the long-term stability analysis as this is not the main focus of this study.
